# Biochemical and structural basis for differential inhibitor sensitivity of EGFR with distinct exon 19 mutations

**DOI:** 10.1038/s41467-022-34398-z

**Published:** 2022-11-10

**Authors:** Iris K. van Alderwerelt van Rosenburgh, David M. Lu, Michael J. Grant, Steven E. Stayrook, Manali Phadke, Zenta Walther, Sarah B. Goldberg, Katerina Politi, Mark A. Lemmon, Kumar D. Ashtekar, Yuko Tsutsui

**Affiliations:** 1 Department of Pharmacology, Yale University School of Medicine, New Haven, CT 06520 USA; 2 Yale Cancer Biology Institute, Yale University West Campus, West Haven, CT 06516 USA; 3 Yale Cancer Center, Yale University School of Medicine, New Haven, CT 06520 USA; 4 Department of Medicine (Medical Oncology), Yale School of Medicine, New Haven, CT 06520 USA; 5 Yale Center for Analytical Sciences, Yale School of Public Health, New Haven, CT 06520 USA; 6 Department of Pathology, Yale University School of Medicine, New Haven, CT 06520 USA

**Keywords:** Kinases, Non-small-cell lung cancer, X-ray crystallography, Cancer therapeutic resistance

## Abstract

Tyrosine kinase inhibitors (TKIs) are used to treat non-small cell lung cancers (NSCLC) driven by epidermal growth factor receptor (EGFR) mutations in the tyrosine kinase domain (TKD). TKI responses vary across tumors driven by the heterogeneous group of exon 19 deletions and mutations, but the molecular basis for these differences is not understood. Using purified TKDs, we compared kinetic properties of several exon 19 variants. Although unaltered for the second generation TKI afatinib, sensitivity varied significantly for both the first and third generation TKIs erlotinib and osimertinib. The most sensitive variants showed reduced ATP-binding affinity, whereas those associated with primary resistance retained wild type ATP-binding characteristics (and low *K*_M, ATP_). Through crystallographic and hydrogen-deuterium exchange mass spectrometry (HDX-MS) studies, we identify possible origins for the altered ATP-binding affinity underlying TKI sensitivity and resistance, and propose a basis for classifying uncommon exon 19 variants that may have predictive clinical value.

## Abstract (web summary)

Although small molecule tyrosine kinase inhibitors are effective in lung cancer driven by mutated EGFR, some receptor variants fail to respond. Here, the authors identify structural features of an important set of EGFR variants with reduced inhibitor sensitivity, guiding future inhibitor selection.

## Introduction

**Fig. 1:**
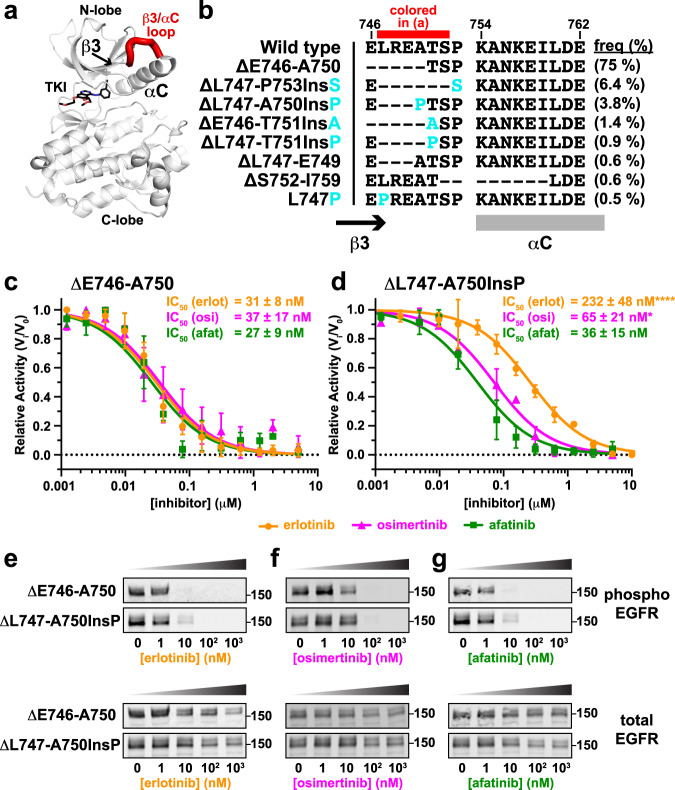
Differential inhibitor sensitivity of purified EGFR TKDs. **a** Crystal structure of the EGFR kinase domain bound to erlotinib (PDB ID: 1M17^40^), showing the location of the β3-αC loop (red) encoded within *EGFR* exon 19. Erlotinib is shown in black sticks. **b** Sequences of the β3/αC region of wild type EGFR and each exon 19 variant that could be studied here (see Supplementary Fig. 1), with deleted residues indicated by dashes and inserted non-native amino acids highlighted in cyan. The reported frequency of each exon 19 variant in the COSMIC database^3^—as a percentage of all exon 19 mutations seen in NSCLC patients—is shown in parenthesis. **c**, **d** Concentration dependence of inhibition of 100 nM purified EGFR TKD containing the ΔE746-A750 (**c**) or ΔL747-A750InsP (**d**) mutation with erlotinib (orange), osimertinib (magenta) or afatinib (green) in the presence of 1 mM ATP, 10 mM MgCl_2_, and 10 μM peptide substrate at 30 °C (see “Methods”). IC_50_ was obtained by fitting data to the equation: Rate = 100/(1+[TKI]/IC_50_), and mean values (± SD) are quoted for *n* = 3 separate protein preparations, with three independent experiments for each. Error bars on all points represent SD across all independent experiments. *P* values from unpaired two-sided Student’s t-tests suggest that IC_50_ values for erlotinib (*P* < 0.0001) and osimertinib (*P* = 0.03) are significantly increased for ΔL747-A750InsP (*n* = 6). **e**–**g** LICOR Western blots for total cell lysates from CHO cells transfected with full-length EGFR constructs harboring the ΔE746-A750 deletion or ΔL747-A750InsP indel, treated with erlotinib (**e**), osimertinib (**f**) or afatinib (**g**) at the concentrations noted for 1 h prior to lysis. Blots were probed with antibody against phosphotyrosine 1173/1197 in EGFR (upper pair) and total EGFR (lower pair), both visualized simultaneously using a LICOR imaging system. Position of the 150 kDa molecular weight marker is shown. To compare inhibition of ΔE746-A750 and ΔL747-A750InsP by a given TKI, the two exposures are matched so that total signal intensity in the DMSO-treated band is the same for both variants. Source data and uncropped gels (including Grb2 control blots) are provided as a Source data file.

Almost two decades since the discovery of activating mutations in the tyrosine kinase domain (TKD) of the epidermal growth factor receptor (EGFR) that drive non-small cell lung cancer (NSCLC)^1^, tyrosine kinase inhibitors (TKIs) are now used routinely as the first-line treatment to target EGFR with significant clinical benefit. The most frequently observed *EGFR* mutations in NSCLC patients, together accounting for over 90% of oncogenic *EGFR* aberrations in this cancer^2–4^, are in exons 19 (45%^4^) and 21 (46%^4^). The most common exon 19 mutation, which accounts for ~33% of *EGFR* mutations in NSCLC, causes a 5-residue deletion (ΔE746-A750) from the loop connecting the third β-strand of the EGFR TKD (β3) to the key regulatory αC helix (Fig. 1a). The most common exon 21 mutation is L858R in the TKD activation loop, which accounts for 44% of *EGFR* mutations in this cancer^4^. Five TKIs (erlotinib, gefitinib, afatinib, dacomitinib, and osimertinib) have been approved by the FDA for the treatment of *EGFR*-mutated NSCLC with exon 19 deletions or an L858R mutation^5^, with first generation TKIs such as erlotinib previously recommended as optimal first-line treatment^6^. Unfortunately, development of resistance to these TKIs is very common, most frequently through emergence of the T790M mutation in exon 20^7^. Efforts to improve patient outcomes led current National Comprehensive Cancer Network clinical practice guidelines to suggest the third generation irreversible TKI osimertinib for first-line treatment of EGFR-mutated NSCLC^8^.

The Catalogue of Somatic Mutations in Cancer (COSMIC)^3^ lists over 100 different exon 19 mutations (including many singletons) in lung cancer, but the predominance of the ΔE746-A750 variant (Fig. 1b)—which accounts for 75% in this database—has led current clinical recommendations to consider them all as a single group. Numerous studies have reported differential sensitivity of individual EGFR exon 19 variants to EGFR TKIs in clinical settings, however^9–23^. For example, in a cohort of 202 patients with tumors harboring various exon 19 deletions, uncommon deletion variants were associated with significantly worse survival than the common ΔE746-A750 mutation with the first generation TKI gefitinib^24^. In addition, our group previously reported significantly shorter progression-free and overall survival in erlotinib-treated patients with tumors carrying the ΔL747-A750InsP mutation compared to those with tumors driven by the common ΔE746-A750 mutation^12^. Moreover, the L747P exon 19 mutation has been reported to be associated with primary resistance to first and third generation TKIs^21,25,26^, but to show a significantly better response and progression-free survival to the second generation TKI afatinib^27^. These and other clinical findings argue for the importance of understanding the molecular basis for the differential TKI sensitivity of exon 19-mutated variants.

Here, we describe biochemical and structural studies of EGFR exon 19 variants seen in NSCLC, with a focus on their TKI sensitivities. We previously described differential sensitivity of the common ΔE746-A750 variant and the uncommon ΔL747-A750InsP variant^12^. We recapitulate this observation biochemically and find that it reflects differences in ATP-binding affinity (and *K*_M, ATP_) of the two variants, without measurable alterations in the inhibition constant (*K*_I_) for erlotinib. By comparing a series of different exon 19-mutated variants, we show that they fall into two categories. The common (ΔE746-A750) variant epitomizes a group of variants with reduced ATP-binding affinity that are sensitized to first and third generation inhibitors. The uncommon ΔL747-A750InsP variant exemplifies a distinct group in which ATP-binding affinity (and *K*_M, ATP_) remains at wild type levels, reducing sensitivity to erlotinib and osimertinib. Importantly, we find that afatinib sensitivity is relatively unchanged between the two profiles, which has clinical implications. Determining the first crystal structure of an exon 19-mutated EGFR TKD and analyzing structural dynamics using hydrogen–deuterium exchange mass spectrometry (HDX-MS) further suggested explanations for the different ATP-binding characteristics of exon 19 variants. This in turn allowed us to predict that exon 19 variants with β3/αC loop deletions of ≤3 residues will show primary resistance to first and third generation TKIs. Analysis of outcomes in erlotinib-treated patients was consistent with this prediction, which may be valuable for guiding future clinical decisions.

## Results

### Isolated kinase domains recapitulate exon 19 variant TKI sensitivities

In an effort to understand the biochemical basis for the differential TKI sensitivities of EGFR exon 19 variants, we first asked whether purified EGFR tyrosine kinase domains (TKDs) recapitulate the reduced erlotinib sensitivity seen in cells and patients for the ΔL747-A750InsP variant^12^. We expressed and purified isolated EGFR TKDs harboring either the common exon 19 deletion (ΔE746-A750) or the uncommon ΔL747-A750InsP mutation using a baculovirus expression system (numbers here correspond to UniProt entry P00533, and are 24 greater than those in mature EGFR protein). We used a continuous fluorescence-based assay^28,29^ (see Methods) to monitor peptide phosphorylation by TKDs at 100 nM and ATP at 1 mM in the presence of different EGFR inhibitors. As shown in Fig. 1c, d, significantly higher concentrations of erlotinib (*P* < 0.0001) and osimertinib (*P* = 0.03) were required for 50% inhibition (IC_50_) of ΔL747-A750InsP kinase activity (Fig. 1d) than of ΔE746-A750 (Fig. 1c). As in previous studies^12^, the increase in IC_50_ was greater for erlotinib (~7.5-fold) than for osimertinib (~2-fold increase in IC_50_), whereas the increase in IC_50_ for afatinib was only ~1.3-fold and was not statistically significant (*P* = 0.24). As in our previously published study^12^, similar differences were also observed when assessing inhibition of intact EGFR variants expressed in CHO cells (right panels in Fig. 1e–g)—although the present studies did indicate slightly reduced afatinib sensitivity for ΔL747-A750InsP in CHO cells, unlike our previous work. IC_50_ values cannot be directly compared across inhibitors in these studies, since erlotinib is a reversible inhibitor whereas osimertinib and afatinib are both irreversible covalent inhibitors.

### In vitro quantitation of inhibitor sensitivity of other exon 19 variants

Having recapitulated the previously-observed differences between ΔE746-A750 and ΔL747-A750InsP EGFR variants with in vitro biochemical studies, our next goal was to extend this analysis to other unstudied exon 19 variants. We generated expression constructs for an additional 13 different exon 19 variants (see Supplementary Fig. 1a), focusing on those that occur most frequently in lung cancer^11,12^. Unfortunately, about half of these variants expressed poorly or aggregated during purification; this likely explains why no crystal structure has yet been reported for EGFR’s TKD with an exon 19 deletion. Nonetheless, we could generate high quality TKD protein for six further exon 19 variants (Supplementary Fig. 1b, c), including TKD containing the L747P mutation—reported to be associated with clinical resistance to erlotinib and gefitinib^21,25,26^. Figure 1b lists the mutations in the β3/αC loop (Fig. 1a, b) that we could study biochemically, as well as their frequencies among exon 19 variants in the COSMIC database^3^. Kinetic studies showed that most of the activated exon 19 TKD variants had apparent *k*_cat_ values ranging from 0.5 to 1.2 s^−1^ (Table 1)—compared with <0.05 s^−1^ for wild type and 1.4 s^−1^ for the L858R-mutated TKD, in agreement with previous reports^30,31^. Both ΔE746-T751InsA and ΔL747-T751InsP had lower apparent *k*_cat_ values (0.12 and 0.16 s^−1^ respectively). This presumably reflects smaller proportions of active protein in these preparations, given the very low values for afatinib IC_50_ determined in these cases. As one approach to correct for this effect, we assumed that IC_50_ for afatinib (see below) under these conditions is equal to 50% of the active TKD concentration, and corrected *k*_cat_ accordingly (right-most column in Table 1). The resulting corrected *k*_cat_ values range from 0.75 to 1.33 s^−1^ with two outliers. ΔS752-I759 appears approximately two-fold more active, and the ΔL747-E749 variant did not show elevated kinase activity (Table 1), which was unexpected.

**Table 1:** Biochemical properties of EGFR TKDs with different mutations

Mutation	IC_50_ erlotinib (nM)	IC_50_ afatinib (nM)	IC_50_ osimertinib (nM)	*K*_I_ erlotinib (nM)	*K*_M, ATP_ (μM)	*K*_M, pept_ (μM)^b^	apparent *k*_cat_ (s^−1^)^b^	corrected *k*_cat_ (s^−1^)^b,c^
None (wild type)	n.d.	n.d.	n.d.	n.d.	12 ± 3	406	0.042	0.042
**Profile 1**								
ΔL747-A750InsP	232 ± 48	36 ± 15	65 ± 21	5.3 ± 1.5	23 ± 6	15	0.91	1.26
L747P	141 ± 17	37 ± 4	159 ± 89	6.2 ± 4.0	21 ± 6	52	0.84	1.14
ΔL747-E749	108 ± 17^a^	42 ± 4^a^	>1000^a^	n.d.	13 ± 8	n.d.	~0.044	0.052
**Profile 2**								
ΔE746-A750	31 ± 8	27 ± 9	37 ± 17	n.d.	158 ± 24	261	0.60	1.11
ΔE746-T751InsA	20 ± 3	8 ± 1	11 ± 2	n.d.	240 ± 59	156	0.12	0.75
ΔL747-T751InsP	14 ± 5	6 ± 2	7 ± 2	n.d.	164 ± 48	38	0.16	1.33
ΔL747-P753InsS	37 ± 10	23 ± 3	26 ± 16	4.2 ± 1.7	101 ± 22	292	0.49	1.07
ΔS752-I759	25 ± 5	20 ± 7	22 ± 7	n.d.	91 ± 25	80	1.2	3.0
**Other**								
L858R	75 ± 12	23 ± 2	52 ± 2	4.7 ± 2.3	74 ± 14	218	1.4	3.04
L858R/T790M	~10,000	92 ± 23	101 ± 24	41 ± 14	45 ± 6	99	6.6	-

^a^IC_50_ determinations for ΔL747-E749 were performed at 1 μM TKD, whereas experiments with all other variants were performed at 100 nM TKD. IC_50_ values for ΔL747-E749 have therefore been divided by 10.

^b^Titration to maximum peptide concentration was only performed once, because of the large amounts of peptide required, so *K*_M, pept_ and apparent *k*_cat_ values are estimates with no S.D. quoted (*n* = 1).

^c^As afatinib is highly potent and its IC_50_ is frequently less than 50% of the TKD concentration, we assumed an endpoint titration to estimate the active TKD concentration (equal to afatinib IC_50_, which we assume is 50% of active [TKD]), and have corrected the apparent *k*_cat_ based on this number. L858R/T790M was excluded from this analysis.

**Fig. 2:**
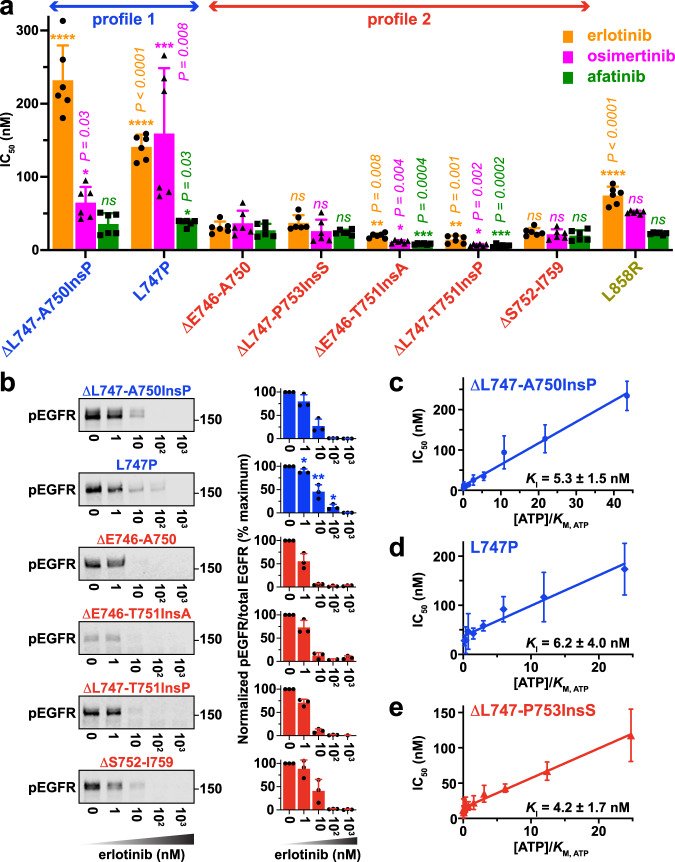
Quantitation of in vitro inhibitor sensitivity of other EGFR exon 19 variants. **a** Bar graph of IC_50_ values for erlotinib (orange), afatinib (green), and osimertinib (magenta) obtained from dose-response curves using purified TKD as described in Methods. All IC_50_ values represent means (± SD) for *n* = 3 separate protein preparations, with two independent experiments for each. Asterisks denote whether IC_50_ is significantly different for a given TKI (erlotinib, osimertinib or afatinib) when compared (pairwise) with the value obtained for ΔE746-A750. *P* values from unpaired two-sided Student’s t-tests are given when <0.05 (*P < 0.05; **P < 0.01; ***P < 0.001, ****P < 0.0001). Based on these IC_50_ values, we classify the exon 19 variants into two profiles: Profile 1 (Blue: resembling ΔL747-A750InsP) or Profile 2 (Red: resembling ΔE746-A750) —see also Supplementary Fig. 2. **b** pEGFR immunoblots of total cell lysates from CHO cells transfected with full-length EGFR constructs harboring the indicated exon 19 mutations. Cells were treated with erlotinib at indicated concentrations for 1 h prior to lysis, and blots probed with anti-pY1173/1197. Position of the 150 kDa molecular weight marker is given. The bar graphs at the right of each blot plot normalized mean pEGFR signal relative to total EGFR signal as a percentage of maximum receptor phosphorylation (± SD for *n* = 3 biological replicates). Asterisks denote statistically significant differences from ΔE746-A750, seen for L747P at 1 nM erlotinib (*P* = 0.0271), 10 nM erlotinib (*P* = 0.01), and 100 nM erlotinib (*P* = 0.0328) from unpaired two-sided Student’s t-tests. **c**–**e** Cheng-Prusoff plots of mean values (± SD) for determining inhibition constants (*K*_I_) for erlotinib with purified ΔL747-A750Ins P (**c**), L747P (**d**), and ΔL747-P753InsS (**e**) TKDs (see Methods), with *K*_I_ values listed as mean (± SD) for *n* = 3 separate protein preparations. See also Supplementary Fig. 3. Source data and uncropped gels (including total EGFR and Grb2 loading controls) are provided in the Source data file.

We next analyzed inhibition of the exon 19 variants (at 50–100 nM protein) by erlotinib, osimertinib, and afatinib (at 1 mM ATP). IC_50_ values corresponded to ~50% of active TKD concentration in most cases (~20–40 nM), suggesting very high affinity inhibitor binding—although the ΔL747-T751InsP and ΔE746-T751InsA variants gave lower values (indicating that these preparations have reduced levels of active kinase). Whereas this high sensitivity to afatinib was maintained across all exon 19 variants studied (green bars in Fig. 2a), a subset of variants showed significantly reduced sensitivity to erlotinib and osimertinib (Fig. 2a; see also Table 1 and Supplementary Fig. 2). Thus, two distinct sensitivity classes or profiles could be defined. In the first (profile 1: Fig. 2a), which comprises ΔL747-A750InsP and L747P, sensitivity to erlotinib and osimertinib was substantially reduced (IC_50_ increased) compared with ΔE746-A750. In profile 2 variants, by contrast, sensitivity to erlotinib and osimertinib was more similar to that seen for the common ΔE746-A750 variant (Fig. 2a). The L858R control appears to be intermediate between the two profiles. L858R is slightly less sensitive to erlotinib than ΔE746-A750 (by ~2.5-fold)—consistent with previous reports^32,33^. It is important to note that we cannot directly compare sensitivity across different inhibitors without time-dependent inhibition studies.

The reduced erlotinib sensitivity of profile 1 variants was also discernible for mutated full-length receptors expressed in CHO cells (Fig. 2b)—with L747P showing the effect most clearly, consistent with clinical reports^21^. We also investigated another rare exon 19 deletion variant, ΔL747-E749, which has 33 entries in COSMIC^3^. Unexpectedly, we were unable to express the intact receptor with this mutation in CHO cells, and the purified TKD was much less active in vitro than TKDs with the other mutations listed in Fig. 1b. When corrected for the fact that experiments with this variant required 10-fold more TKD, estimated IC_50_ values for ΔL747-E749 were 108 nM for erlotinib, 42 nM for afatinib, and >1000 nM for osimertinib (Supplementary Fig. 2f, Table 1)—placing it in profile 1.

These data demonstrate that sensitivity to a given inhibitor can differ significantly between individual EGFR exon 19 variants at the level of the kinase domain itself. Most notably, and consistent with recent clinical data^17,21,25^, we find that relative resistance to erlotinib is seen with L747P and ΔL747-A750InsP (in profile 1)—but remarkably not with ΔL747-T751InsP (in profile 2), which differs solely in having just one more amino acid deleted from its β3/αC loop.

### Profile 1 and profile 2 variants have similar drug-binding affinities

We next wanted to understand the origin of different inhibitor sensitivities of EGFR exon 19 deletion variants. We undertook steady state kinetic measurements of TKD activities to measure their Michaelis constants for ATP (*K*_M, ATP_) and, where possible, inhibition constants (*K*_I_) for erlotinib using the Cheng–Prusoff equation^34^. We were able to obtain reliable *K*_I_ estimates for the ΔL747-A750InsP, L747P, and ΔL747-P753InsS variants (Fig. 2c–e), which all gave values between 4.2 nM (ΔL747-P753InsS) and 6.2 nM (ΔL747-A750InsP), similar to previously reported values^32^. Importantly, the erlotinib *K*_I_ values for the profile 1 ΔL747-A750InsP and L747P variants were both within 1.3–1.5 fold of *K*_I_ for the profile 2 variant ΔL747-P753InsS, despite their erlotinib IC_50_ values being 4–6 fold higher (Fig. 2a). The small differences observed in *K*_I_ when compared with ΔL747-P753InsS are statistically significant (*P* = 0.02 for L747P and *P* = 0.01 for ΔL747-A750InsP). However, they are not sufficient to explain the reduced erlotinib sensitivity of these two profile 1 variants, arguing that reduced drug-binding affinity cannot fully explain resistance to erlotinib—contrary to the suggestions of our previous computational studies^12^.

Equivalent experiments with L858R- and L858R/T790M-mutated TKDs (Supplementary Fig. 3a, b) gave *K*_I_ values of 4.7 ± 2.3 and 41 ± 14 nM respectively, indicating that some of the erlotinib resistance caused by the T790M mutation must arise from reduced drug-binding affinity (increasing *K*_I_)—consistent with previous studies^30,31,33^. Unexpectedly, Cheng–Prusoff equation plots for other profile 2 variants showed a lack of dependence of IC_50_ on ATP concentration over a substantial range (Supplementary Fig. 3c, d), suggesting either non-competitive inhibition effects that are difficult to understand and/or non-specific binding of inhibitor to inactive protein in these preparations. As a result, we could not estimate *K*_I_ values for the ΔE746-A750, ΔE746-T751InsA, ΔL747-T751InsP or ΔS752-I759 variants.

### TKI sensitivity is determined by *K*_M, ATP_

**Fig. 3:**
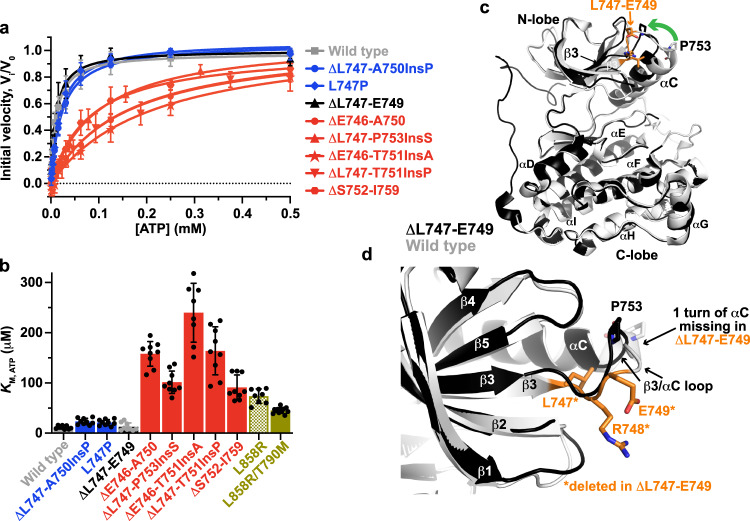
TKI sensitivity of exon 19 variants is determined by *K*_M, ATP_. **a** Michaelis-Menten plots for the indicated EGFR kinase domains in the presence of 20 μM peptide substrate (*n* = 3 separate protein preparations, with three independent experiments for each) with the TKD concentrations listed in Methods. Normalized mean initial velocity data were plotted against ATP concentration (error bars represent SD across replicates) and fit to the Michaelis–Menten equation to obtain values for *K*_M, ATP_. **b** The mean value of *K*_M, ATP_ obtained by fitting data from individual ATP concentration series is plotted (± SD). The bar for wild type kinase is grey, profile 1 variants in blue, profile 2 variants in red, L858R variants in olive, and the unactivated ΔL747-E749 variant in black. Data represent three separate protein protein preparations, with three independent experiments for each. **c** Overlay of the 2.96 Å crystal structure of the ∆L747-E749 exon 19 variant (PDB ID: 7TVD – see Table 2), shown in black, with the wild type EGFR TKD from PDB entry 1M14^40^, shown in grey. Both TKDs are in the active conformation, but the deletion in the β3/αC loop displaces αC slightly towards the ATP-binding site in ∆L747-E749 (see green arrow). The residues deleted in ∆L747-E749 (L747, R748, and E749) are shown in orange in the wild type structure. **d** Close-up of the β3/αC region in the overlay of ΔL747-E749 on the wild type EGFR TKD, showing that β3 is slightly truncated at its C-terminus in ΔL747-E749 and αC loses a helical turn at its N-terminus—allowing the shortened β3/αC loop to link the two elements. Source data are provided as a Source data file.

Previous studies of acquired TKI resistance in EGFR^30,33^, ABL^35^—as well as primary resistance in ALK^36^—have shown that alterations in ATP-binding affinity play a determining role. Measurements of *K*_M, ATP_ for all the EGFR variants listed in Fig. 1b revealed that this is also true for exon 19 mutations in EGFR. Remarkably, the profile 1 and profile 2 variants also cluster in the same groups based on their *K*_M, ATP_ values (Fig. 3a, b). Notably, *K*_M, ATP_ for ΔL747-A750InsP is ~seven-fold lower (23 ± 6 nM) than the value of 158 ± 24 nM measured for ΔE746-A750 (*P* < 0.0001). This difference in *K*_M, ATP_ can almost fully account for the 7.5-fold difference in erlotinib IC_50_ between the two variants. Similarly, *K*_M, ATP_ for the L747P variant (7.5-fold smaller than for ΔE746-A750; *P* < 0.0001) can more than account for its ~five-fold reduced sensitivity to erlotinib. All the exon 19 variants in profile 2 have higher *K*_M, ATP_ values—ranging from 91 to 240 μM (Table 1)—all of which are greater than the *K*_M, ATP_ value that we measured for L858R-mutated TKD (74 µM).

These data suggest that—as with L858R—most activating exon 19 mutations reduce ATP binding affinity (higher *K*_M, ATP_), resulting in enhanced sensitivity to ATP-competitive inhibitors like erlotinib. The increase in *K*_M, ATP_ value appears to be greater for these exon 19 mutations than for L858R, suggesting that they may promote higher sensitivity to TKIs than L858R—possibly explaining differences in outcome between these two groups first described in early clinical studies^37,38^. By contrast, the profile 1 exon 19 variants (L747P and ΔL747-A750InsP) activate EGFR without increasing *K*_M, ATP_ (Table 1), and retain the ATP-binding characteristics of the wild type kinase and the ΔL747-E749 variant (Fig. 3b). This is associated with lower TKI sensitivity (and clinical resistance^12^). As previously reported by Eck and colleagues^30^, the T790M mutation reverses the *K*_M, ATP_ increase caused by the L858R mutation to reduce TKI sensitivity. However, T790M does not fully bring *K*_M, ATP_ down to wild type levels under our assay conditions (Fig. 3b and Supplementary Fig. 3e)^30,31^, and the *K*_M, ATP_ change is not sufficient to account for the >130-fold increase in IC_50_ for erlotinib (Supplementary Fig. 2g, h) seen for the L858R/T790M double mutation. Taken together, our findings with exon 19 variants suggest that, just as suggested for T790M^30^, alterations in ATP-binding affinity are the primary (if not sole) cause of reduced sensitivity to erlotinib and osimertinib.

### Crystal structure of ΔL747-E749 EGFR TKD

Despite significant efforts, we were unable to crystallize any of the activated exon 19 EGFR TKD variants listed in Fig. 1b. We did succeed in determining a 3 Å crystal structure of the ΔL747-E749 variant, however (PDB ID 7TVD, see Supplementary Fig. 4 and Table 2). This variant was indistinguishable in kinetic assays from wild type (Table 1), unexpectedly failing to show elevated kinase activity. The ΔL747-E749 variant crystallized in the same space group as the wild type EGFR TKD^39,40^, and formed the same asymmetric dimer reported to stabilize the active kinase conformation in crystals of other EGFR TKDs. As shown in Fig. 3c, d, the only discernible differences between the wild type TKD and ΔL747-E749 are a shortening of strand β3 (from its C-terminal end) and loss of the first turn of helix αC. These changes result from deletion of the three residues (L747, R748, and E749: orange in Fig. 3d) from the beginning of the β3/αC loop. Truncation of both β3 and αC is necessary to allow the shortened (5-residue) β3/αC loop in the ΔL747-E749 variant still to connect these two secondary structure elements, aided by a slight displacement of αC towards the ATP-binding site compared with its position in dimers of wild type, L858R or other variants with activating mutations (green arrow in Fig. 3c). The predicted salt bridge between E762 in αC and K745 in strand β3, required to stabilize ATP interactions of the latter in the active TKD, is precisely maintained in ΔL747-E749 (Supplementary Fig. 4). The ΔL747-E749 TKD structure also shows that a 3-residue deletion from the β3/αC loop can be tolerated without major disruption. AlphaFold2^41^—based predictions performed using ColabFold^42^ suggested that longer deletions in profile 2 variants (notably ΔS752-I759) further truncate αC from its amino-terminus to allow the loop still to reach between the β3 and αC secondary structure elements (Supplementary Fig. 5). This likely leads to more profound distortion and/or alterations in αC position and interactions—consistent with our failure to crystallize such exon 19 variants. The profile 1 variants have less truncated αC helices.

**Table 2:** Crystallization conditions, data collection, and refinement statistics

Amino acid residue boundaries	696–1022
PBD ID	7TVD
Crystallization conditions	Protein in 20 mM Tris pH 8.0, 150 mM NaCl and 2% glycerol: Reservoir solution 28% PEG 400, 0.1 M HEPES pH 7.5, 0.2 M CaCl_2_. Ratio = 1:1, 16 °C
*Data collection*^*a*^	
Beamline	APS/NE-CAT 24-ID-C
Date of collection	March 19, 2019
Wavelength (Å)	0.97918
Space group	*I 2 3*
*Cell dimensions*	
*a*, *b*, *c* (Å)	149.42, 149.42, 149.42
α, β, γ (°)	90.00, 90.00, 90.00
Resolution (Å)	105.66–2.96
Completeness	99.9 (99.7)
Redundancy	36.1 (33.5)
*R*_sym_ (%)	44 (263)
*I*/*σ*	15.4 (1.5)
CC^1/2^	0.999 (0.44)
Number of reflections	11,749 (1880)
*Refinement*	
*R*_work_/*R*_free_ (%)	24.0/27.7
*Number of atoms*	
Protein	2338
Ions	0
Ligands	0
Water	0
*Average B factor (Å)*	
Protein	104.0
Ions	-
Ligands	-
Water	-
*Geometry (Ramachandran)*	
Favored (%)	94.06
Allowed (%)	5.24
Outliers (%)	0.70
*RMSD (Å)*	
Bond length	0.004
Bond angle	0.77

^a^Numbers in parentheses denote highest resolution shell.

It is not clear from this analysis why the ΔL747-E749 TKD is not activated whereas ΔL747-A750InsP and L747P are both among the most active of the variants (Table 1). These two activated variants have slight amino-terminal truncations of αC compared with ΔL747-E749 in AlphaFold models (Supplementary Fig. 5a), but share the introduction of a proline at position 747 (two residues after the β3 lysine). This could alter local dynamics in a way that mimics a larger deletion to activate the kinase.

### HDX-MS shows variant-specific differences in structural dynamics

**Fig. 4:**
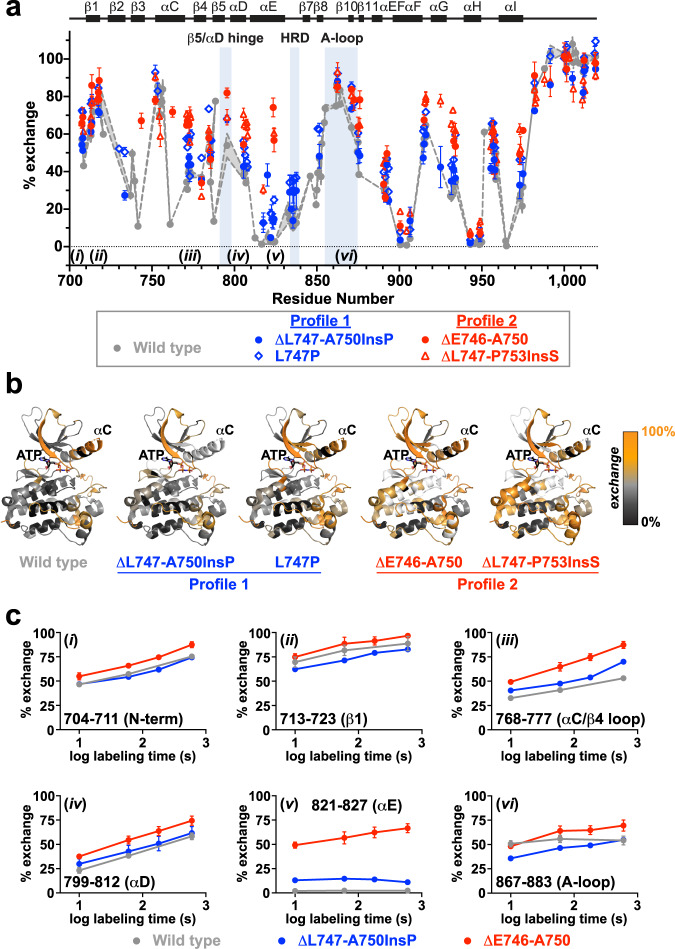
Native state dynamics of exon 19 variants differ for profile 1 and profile 2. **a** Backbone amide hydrogen exchange from HDX-MS experiments for all analyzable peptides at the 1 min labeling timepoint for wild type (grey circles), ΔL747-A750InsP (blue circles), L747P (blue diamonds), ΔE746-A750 (red circles), and ΔL747-P753InsS (red triangles) variants without ATP or inhibitor. Mean percent exchange (± SD) for three independent experiments across each of three separate protein preparations (two for ΔL747-A750InsP) is plotted against the median residue number of the peptide in wild type EGFR numbering. Secondary structure element positions are denoted at the top of the figure, with functionally important regions (β5/αD hinge, HRD motif, and activation loop) indicated. Wild type data are represented by two dashed grey lines corresponding to the range (± SD) for each point. The ΔE746-A750 and ΔL747-P753InsS variants both showed EX1 kinetics^48^ in the HRD and DFG motif regions, so these data are shown separately (see Fig. 6). Positions of peptides *i* through *vi* (see **c**) are denoted at the bottom of the graph. **b** 1 min percent exchange data for the indicated EGFR variants mapped onto a crystal structure of wild type EGFR kinase domain in its active conformation (PDB ID: 7KXZ), with ATP (sticks) placed based on its position in PDB ID: 3VJO^45^. The percent exchange of each amino acid was assigned using the DynamX software package (Waters) as described in Methods, and is color coded as shown on a scale from orange (100% exchange) to dark grey (no exchange). White regions in the binding pocket and elsewhere represent regions with EX1 exchange kinetics or with no coverage. Corresponding representations of 10 s and 10 min data are provided in Supplementary Fig. 7. **c** Mean percent exchange data (± SD) for key individual peptides *i* through *vi*, as marked in (**a**), for wild type (grey), ΔL747-A750InsP (blue), and ΔE746-A750 (red) as a function of the logarithm of deuterium labeling time are shown. See Supplementary Fig. 8 for additional peptides. Three independent HDX-MS experiments were performed on each of three separate protein preparations (two for ΔL747-A750InsP), with experimental parameters listed in Supplementary Table 1. Source data are provided as a Source data file.

To gain insight into structural differences between profile 1 and profile 2 exon 19 variants, we used hydrogen–deuterium exchange mass spectrometry (HDX-MS), which probes region-specific structural dynamics by measuring rates of backbone amide hydrogen atom exchange^43,44^. Because amide hydrogens in structurally flexible or solvent-accessible regions exchange with deuterium (present as D_2_O in the solvent) more rapidly than in structurally rigid regions, time-dependent deuterium uptake can be used to assess local backbone flexibility. We compared wild type EGFR TKD with two profile 1 variants (ΔL747-A750InsP and L747P) and two profile 2 variants (ΔE746-A750 and ΔL747-P753InsS). In the absence of erlotinib, all mutated variants showed a generally increased level of backbone amide hydrogen exchange (Fig. 4a, b and Supplementary Figs. 6a, 7) across most regions of the protein. This effect was most notable in the region between helix αC and strand β7 (residues 750–850), which surrounds the binding site for ATP and ATP-competitive TKIs (Fig. 4b^45^)—notably in the αC/β4 loop and αE/HRD motif region. Importantly, profile 2 variants (red in Fig. 4a and Supplementary Fig. 6a) generally showed higher degrees of exchange in these regions than profile 1 variants (blue in Fig. 4a and Supplementary Fig. 6a). Peptides around αG (residue 920) also showed some differences—particularly in ΔL747-P753InsS—that were unexpected given their significant distance from the ATP-binding site. Interestingly, parallel HDX-MS analysis of the less active ΔL747-E749 TKD variant showed that it does not show the enhanced exchange seen for other exon 19 deletion variants (Supplementary Fig. 6c). This finding suggests that increased structural dynamics (as manifest in HDX-MS studies) may play an important role in activation of EGFR by exon 19 deletions.

**Fig. 5:**
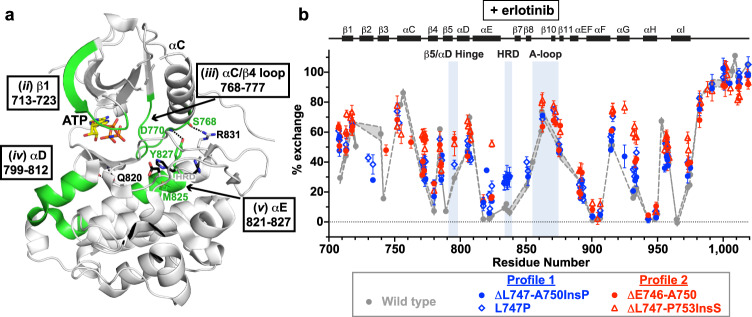
Location of peptides with increased exchange, and dampening by erlotinib. **a** The regions corresponding to peptides *ii*, *iii*, *iv*, and *v* in Fig. 4c are highlighted in green in the structure of wild type EGFR (PDB ID: 7KXZ), with ATP (sticks) positioned as in Fig. 4b. These regions all show increased exchange in profile 2 variants, and surround the ATP-binding site. Interactions between helices αE and αC mediated by the Y827 and R831 side chains and S768 and D770 main chain atoms, allowing αC/αE communication, are shown as dotted lines. Q820, which interacts with the β7/β8 hairpin, is also shown, as is the M825 side chain in αE that contacts the histidine of the catalytic loop’s HRD motif. **b** Percent exchange data in the presence of erlotinib for all analyzable peptides at the 1 min labeling timepoint for wild type (grey points), ΔL747-A750InsP (blue circles), L747P (blue diamonds), ΔE746-A750 (red circles), and ΔL747-P753InsS (red triangles) variants. Erlotinib was present at a 1.4- to 2-fold molar excess (15.6–20 μM, depending on TKD concentration). The figure is labeled as in Fig. 4a. Data are plotted as the mean percent exchange (± SD) from three independent experiments on each of three separate protein preparations (two for ΔL747-A750InsP), with experimental parameters listed in Supplementary Table 1. Peptides with EX1 kinetics are excluded from this figure, and are reported on in Fig. 6. Source data are provided as a Source data file.

In Fig. 4c, HDX data for six individual peptides (i–vi: locations also noted in Fig. 4a) are compared for ΔE746-A750 (red), ΔL747-A750InsP (blue), and wild type (grey). These results illustrate the greater level of exchange for ΔE746-A750 compared with ΔL747-A750InsP in peptides from the β1 strand (ii), αC/β4 loop (iii), αD (iv), αE (v), and the activation loop (vi) regions. The observed differences suggest significantly increased structural flexibility or disorder in the ATP/inhibitor binding site for the most common (ΔE746-A750) exon 19 deletion (profile 2) compared with wild type or the ΔL747-A750InsP (profile 1) variant. Interestingly, the most dramatic difference in exchange was seen for peptides encompassing residues 821–827 (v in Fig. 4c). This corresponds to the C-terminal half of αE in the EGFR TKD (see also Supplementary Fig. 8e, f). Helix αE makes direct contacts with the C-terminal part of helix αC in both active and inactive EGFR TKD structures (Fig. 5a), so would be expected to be altered when the stability or dynamics of αC are changed. As shown in Fig. 5a, the Y827 side chain (in helix αE) forms a predicted hydrogen bond with the backbone amide of D770 (close to the C-terminus of αC, and the site of activating exon 20 insertions^2^). The R831 side chain (at the very end of αE) also interacts with the S768 main chain in αC (Fig. 5a). Helix αC changes that result from profile 2 exon 19 deletions between β3 and αC are presumably communicated to αE through these interactions, which interestingly were also recently implicated in differential gefitinib sensitivity in molecular dynamics studies^46^. The altered αE peptide also includes M825, the side chain of which contacts H835 in the conserved HRD motif at the beginning of the TKD’s catalytic loop—propagating αC changes to the catalytic loop. Changes in αE dynamics also appear to be propagated to αD, which plays a key role in stabilizing the ATP-binding site (Fig. 5a). In addition, the Q820 side chain adjacent to this peptide appears to help stabilize the β7/β8 hairpin that contributes to ATP binding. Other peptides that show the most elevated exchange in ΔE746-A750 also lie adjacent to the ATP-binding site (Fig. 5a), suggesting that their structural fluctuations seen by HDX-MS reflect disorder in this binding site caused by the deletion between β3 and αC. The profile 2 variant ΔL747-P753InsS also shares these dynamic properties with ΔE746-A750, whereas the L747P variant appears intermediate. Exchange plots are compared for selected peptides captured for all (or most) variants studied in Supplementary Fig. 8, and also illustrate the generally increased exchange seen in different regions of the TKD for profile 2 compared with profile 1 exon 19 variants.

When HDX-MS experiments were repeated in the presence of erlotinib, the differences outlined above were much less evident (Fig. 5b and Supplementary Fig. 6b, d), and the dynamics of the erlotinib-bound exon 19 variants were similar to those of erlotinib-bound wild type TKD. As expected, therefore, erlotinib binding causes structural rigidification of exon 19 mutants, and the conformational space sampled by the erlotinib-bound variants is narrowed so that it is no longer significantly different from that of the wild type TKD. One exception is ΔL747-P753InsS, which shows more exchange than the other erlotinib-bound variants in several regions (Fig. 5b), notably around αG.

### ATP-binding pockets of profile 2 variants unfold on minute timescales

**Fig. 6:**
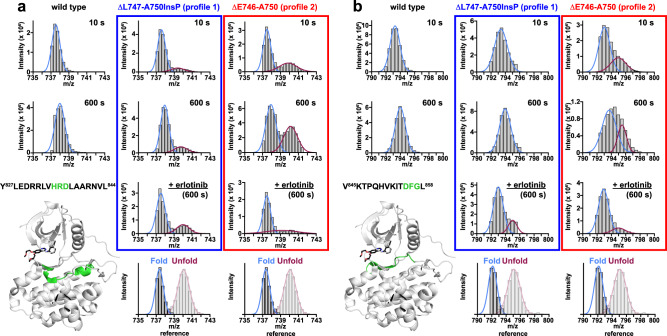
HRD and DFG motif peptides of profile 2 variants are in unfolded regions. **a** Mass spectra of a triply charged peptide (MH^+^ = 2209.2312 Da) containing the HRD motif in wild type (left), ΔL747-A750InsP (blue box), and ΔE746-A750 (red box) at the 10 and 600 s labeling time points. The amino acid sequence of the peptide is shown above the crystal structure (bottom left) and colored green in the structure. The fully deuterated (unfolded) and no deuterium labeling (folded) reference mass spectra are shown below the erlotinib-bound mass spectra. The areas below the light blue and red lines respectively in the bimodal mass envelopes correspond to the protein population within this peptide region (and environment) that is folded and unfolded. Note that erlotinib addition suppressed the unfolded peak in ΔE746-A750 at the 600 s time point. **b** Mass spectra of a doubly charged peptide (MH^+^ = 1582.8952 Da) containing the DFG motif in wild type (left), ΔL747-A750InsP (blue box), and ΔE746-A750 (red box) at 10 and 600 s labeling time points. The peptide region is colored green in the crystal structure at bottom left. Note the bimodal distribution seen for ΔE746-A750, which is collapsed to the folded population upon erlotinib addition. Experimental parameters are given in Supplementary Table 1, and source HDX-MS data are provided as a Source Data file.

One of the most notable features of the HDX-MS data without erlotinib was a bimodal distribution in the exchange kinetics of a few key peptides from profile 2 variants (Fig. 6 and Supplementary Fig. 9). In typical HDX-MS studies of native proteins, the centroid mass of most peptides shows a progressive shift to higher m/Z values over time, in the so-called EX2 limit^47–49^. This reflects frequent but transient/very short-lived local unfolding or structural opening events that refold/close at a higher rate than that of hydrogen/deuterium exchange. By contrast, bimodal mass envelopes are seen in the EX1 limit^48,50^, where the locally unfolded state is much longer lived—reflecting a high energy barrier between the unfolded (exchange-competent) and folded (exchange-incompetent) states, indicating structural heterogeneity. In this EX1 limit, all backbone hydrogens in the peptide either become exchanged simultaneously (high mass envelope) or remain unexchanged (low mass envelope). Bimodal mass envelopes of this sort were seen in both profile 2 variants (Fig. 6 and Supplementary Fig. 9), specifically in peptides from the catalytic loop (which includes the HRD motif) and from the activation loop (which includes the DFG motif). This phenomenon was either not seen, or was much less prominent, in the profile 1 variants studied. These data argue for significant local unfolding of regions around the ATP-binding pocket on the minute timescale – but only for profile 2 and *not* profile 1 exon 19 variants. The fused-bimodal envelopes seen for the DFG-motif region in ΔE746-A750 (Fig. 6b) and ΔL747-P753InsS (Supplementary Fig. 9b) indicate that this region does not fully display EX1 behavior, but again argues for significant disorder in the ATP/drug-binding pocket. Different regions within the same protein or assembly have been reported to display EX1 and EX2 kinetics in several other cases^50–53^. In the EGFR TKD, it appears that the profile 2 mutations cause the appearance of regions with EX1 or EX1-like exchange, which we suggest represents local unfolding of the ATP-binding site.

When erlotinib was added to the ΔE746-A750 TKD, the high mass envelopes of both the HRD and DFG motif peptides were lost (Fig. 6a, b), arguing that drug binding shifts the equilibrium to a folded form—stabilizing the binding pocket and returning it to EX2 kinetics. Interestingly, the same was not true for the ΔL747-P753InsS variant, which still showed a similar bimodal distribution even with erlotinib (Supplementary Fig. 9a, b)—arguing that the erlotinib-bound form of this variant still undergoes significant local unfolding in these regions. This is also consistent with the greater exchange seen across the erlotinib-bound ΔL747-P753InsS TKD in Fig. 5b.

Although the individual exon 19 variants clearly differ from one another, taken together these data argue that exon 19 mutations allosterically modulate the dynamics of the distant ATP-binding pocket to a mutation-specific degree, and that the binding pockets of profile 1 variants (ΔL747-A750InsP and L747P) are more structurally rigid than those of profile 2 variants. The increased disorder or flexibility seen in profile 2 variants appears to correlate with higher *K*_M, ATP_ values, presumably by reducing affinity of the relatively weakly-binding ATP substrate. The tightly-binding inhibitors, however, are presumably less affected by this structural flexibility, as illustrated by the loss of the high mass envelopes for ΔE746-A750 when erlotinib binds in Fig. 6, for example. While the effects are likely to have subtly different structural origins for the different exon 19 deletions, our data suggest that selective impairment of ATP binding through structural disorder may underlie the key difference between the profile 2 variants that are more sensitive to erlotinib and osimertinib and their less sensitive profile 1 counterparts.

### Exon 19 variant features that predict clinical response to erlotinib

The low frequencies of uncommon EGFR exon 19 variants make them challenging to study in statistically-powered clinical trials. It is therefore important to devise predictive methods to guide clinical decision making and trial design—by identifying which uncharacterized variants fall into profile 1 versus profile 2, for example. It is particularly notable that the (profile 1) ΔL747-A750InsP variant shows erlotinib and osimertinib resistance, whereas the (profile 2) ΔL747-T751InsP variant—with just one more β3/αC residue deleted—does not (Fig. 2a). The different TKI sensitivities of these two variants are also supported by recent clinical data^17^, and argue that the proline insertion per se is not a relevant defining factor for whether activated variants are in profile 1 or profile 2. Recently suggested classifications based on where the exon 19 deletion begins^9,16,18^ are also not robust, since we find variants with deletions starting at L747 in both profiles 1 and 2, for example.

The defining characteristic of EGFR exon 19 variants with wild type-like *K*_M, ATP_ values (ΔL747-A750InsP, L747P, ΔL747-E749 and wild type) is that they all have just three or fewer residues missing from the β3/αC loop (Fig. 1b). Our ΔL747-E749 crystal structure (Fig. 3) shows how such a 3-residue deletion can be tolerated without major disruption (consistent with HDX-MS studies of this variant showing a rigid wild type-like ATP-binding site). Introducing a proline at position 747 (as in ΔL747-A750InsP and L747P) seems to be sufficient to activate the kinase (Table 1), with some enhanced structural dynamics (Fig. 4 and Supplementary Fig. 6). Longer deletions—with or without introduction of a proline—require significant structural distortion. Consistent with this, profile 2 variants with longer deletions from the β3/αC loop show increased amide hydrogen exchange in the C-terminal part of helix αC (near the αC/β4 loop—as shown in Supplementary Fig. 8a–c). This disruption is propagated to helices αD, αE (Supplementary Fig. 8d–f) and beyond—to cause substantial disorder in the ATP-binding site as well as TKD activation.

**Fig. 7:**
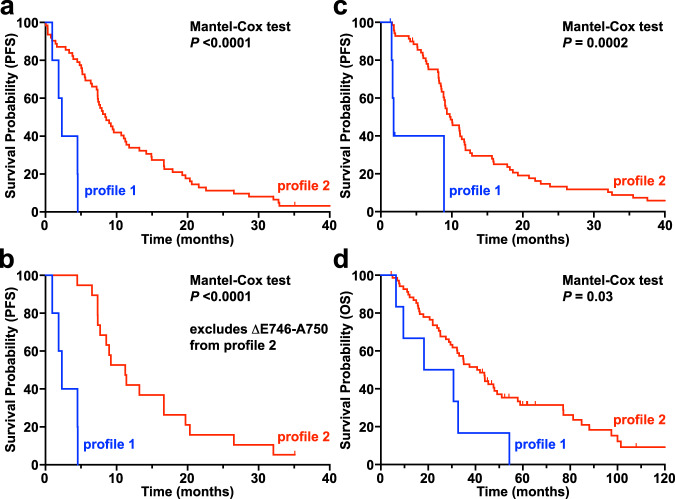
Differential survival of erlotinib-treated profile 1 and profile 2 NSCLC patients. Data for survival outcomes on erlotinib treatment were obtained from the GENIE+ database^54^ (**a**, **b**) and from an institutional cohort (**c**, **d**) as described in the main text for patients with profile 1 (blue) or profile 2 (red) exon 19 mutations. **a** Using GENIE+ data, a profile 1 mutation predicts a significantly shorter median PFS (2.3 months) than a profile 2 mutation (8.5 months) on erlotinib (*P* < 0.0001). We also performed the same analysis excluding the common ΔE746-A750 deletion, since we know this is erlotinib sensitive, and the prediction still holds (**b**), where a profile 1 mutation predicts median PFS of 2.3 months and profile 2 mutation 11.2 months (*P* < 0.0001). Predicted profile 1 mutations in the GENIE+ dataset were restricted to: ΔL747-A750InsP (4 patients) plus ΔE746-L747InsIP (1 patient). Predicted profile 2 mutations were restricted to: ΔE746-A750 (43), ΔL747-P753InsS (5), ΔL747-T751 (3), ΔE746-S752InsV (3), ΔS752-I759 (2), ΔL747-S752 (2), ΔE746-S752InsI (1), ΔE746-T751InsA (1), ΔL747-T751InsP (1), and ΔE746-T751InsIP (1). **c** Similarly, among patients treated with erlotinib at the Yale Cancer Center, a profile 1 exon 19 mutation predicts a significantly shorter median PFS (1.8 months) compared to a profile 2 mutation (9.8 months), with *P* = 0.0002. After adjusting for baseline covariates of age, sex, race, and smoking history using the Cox proportional hazards model^78^, the hazard for progression was nine times greater for patients with tumors with profile 1 compared to profile 2 mutations (95% confidence interval [2.6–31.4], *P* = 0.0005). **d** This holds true for overall survival (OS) on erlotinib as well, with median OS of 24.5 months for profile 1 and 41.3 for profile 2 (*P* = 0.03). The adjusted hazard ratio for death was 3.0 (95% confidence interval [1.2–7.7], *P* = 0.02) for patients with tumors harboring profile 1 mutations compared to profile 2 mutations. Predicted profile 1 mutations in the Yale dataset were restricted to: ΔL747-A750InsP (6 patients). Predicted profile 2 mutations were restricted to: ΔE746-A750 (55), ΔL747-P753InsS (5), ΔL747-T751 (3), ΔL747-T751InsP (2), ΔE746-S752InsV (1), ΔL747-T751InsA (1), ΔL747-S752 (1), ΔE746-T751InsVP (1), ΔT751-I759InsN (1).

Defining profile 1 and profile 2 exon 19 variants as having β3/αC loop deletions of ≤3 or ≥4 residues respectively, we analyzed de-identified outcome data from the GENIE+ database^54^ for patients with a diagnosis of non-small cell lung cancer whose tumors harbored an EGFR exon 19 mutation and were treated with erlotinib. Although the data available for this analysis were very limited, Kaplan–Meier survival analyses (Fig. 7a) showed that a profile 1 mutation is associated with a significantly shorter median progression-free survival (2.3 months) on erlotinib than a profile 2 mutation (8.5 months). Importantly, the association still holds (Fig. 7b) when we reanalyze the data excluding the common ΔE746-A750 deletion (which we know is erlotinib sensitive), with median progression-free survival of 11.2 months for profile 2.

We also performed a similar analysis for a cohort of patients from Yale Cancer Center (Fig. 7c). From March 2005 to December 2021, six patients with tumors with profile 1 EGFR exon 19 mutations and seventy with profile 2 mutations were treated with erlotinib as the first TKI. Baseline characteristics between patients with tumors with profile 1 vs. profile 2 mutations were similar with respect to age (median 60.5 years [range, 50.3–67.8] for profile 1 compared to 62.8 years [36.9–94.5] for profile 2, *P* = 0.70), sex (83.3% female for profile 1 compared to 68.5% for profile 2, *P* = 0.66), and smoking status (66.7% with no prior smoking history for profile 1 compared to 42.8% in profile 2, *P* = 0.39). As with analysis of the GENIE+ data, a profile 1 deletion is associated with a significantly shorter PFS (1.8 months) on erlotinib than a profile 2 deletion (9.8 months). After adjusting for baseline covariates of age, sex, race, and smoking status, the risk of progression was higher for patients with tumors with profile 1 mutations compared to profile 2 (adjusted PFS hazard ratio 9.1 [95% confidence interval, 2.6–31.4, *P* = 0.0005]). Profile 1 mutations are also associated with shorter overall survival on erlotinib (24.5 months) compared to profile 2 mutations (41.3 months), as well as a significantly increased risk of death, with an adjusted overall survival hazard ratio of 3.0 [95% confidence interval, 1.2–7.7, *P* = 0.02] (Fig. 7d).

## Discussion

Recent clinical and laboratory findings—including those presented here—have shown that different EGFR exon 19 variants exhibit different inhibitor sensitivities^9,10,12–14,16,17,19,21–23,25,55^, arguing that they should not be treated as a single group in clinical decision making. Moreover, exon 19 variants can be associated with inhibitor responses that differ from those of other common EGFR mutations found in lung adenocarcinoma^32,38,55,56^. Although the ΔE746-A750 deletion predominates, accounting for 75% of exon 19 variants, more than sixty other different exon 19 aberrations have been detected in lung adenocarcinoma, corresponding to well over 1000 cases in the COSMIC database^3^ alone—so this is not an insignificant problem.

Following our earlier work with the ΔL747-A750InsP EGFR variant^12^, we showed here with in vitro biochemical studies that the origin of the reduced erlotinib sensitivity of this variant arises from its increased ATP-binding affinity (reduced *K*_M, ATP_) compared with the most common exon 19 variant (ΔE746-A750)—impairing erlotinib’s ability to compete with ATP for binding to the TKD’s active site. We found that the L747P variant also shares these characteristics, consistent with reports of its TKI resistance in clinical studies^21,25,26^. Whereas all other activated exon 19 variants that we studied biochemically showed both increased activity and increased *K*_M, ATP_ (by 7.5 to 20-fold) compared with wild type TKD (thus falling into profile 2), two profile 1 variants showed increased activity without any *K*_M, ATP_ alteration (Table 1). Our HDX-MS studies suggest that this difference reflects distinct structural dynamics of profile 1 and profile 2 variants. Profile 2 variants have a disordered ATP-binding pocket (Fig. 6 and Supplementary Fig. 9), correlating with higher *K*_M, ATP_. Profile 1 variants more closely resemble wild type, and bind more strongly to ATP. As a result of these differences, profile 2 mutations simultaneously activate the receptor and sensitize it to ATP-competitive TKIs like erlotinib^32,56^. By contrast, profile 1 variants activate EGFR without simultaneously enhancing TKI sensitivity, and this is manifest as primary resistance to erlotinib and osimertinib, but—importantly—not afatinib (see below). Similar alterations in *K*_M, ATP_ are also known to contribute to acquired TKI resistance associated with the EGFR T790M mutation^30,33^. Moreover, altered *K*_M, ATP_ values can explain primary resistance of ALK-driven neuroblastomas to crizotinib^36,57^. In ALK, one of the most common activating mutations (R1275Q) increases *K*_M, ATP_ by three to five-fold and enhances sensitivity to the ALK inhibitor crizotinib^57^. Another common mutation (F1174L) instead activates without increasing *K*_M, ATP_, and shows no crizotinib response^58^. In both cases, small differences in *K*_M, ATP_ (~3–5-fold) have surprisingly profound consequences in a clinical setting. Our data with exon 19 variants similarly argue that a ~7-fold reduction in *K*_M, ATP_ (Table 1) is sufficient for clinical resistance.

The two activated profile 1 variants studied here (ΔL747-A750InsP and L747P) account for 4.3% of exon 19 aberrations. In addition to these, the COSMIC database^3^ lists around 57 other exon 19 variants that would fall into profile 1 (if activated) based on the length of the β3/αC loop deletion (i.e. with ≤3 residues deleted), as listed in Supplementary Fig. 10. These account for a further ~3.5% of exon 19 variants—placing almost 8% of exon 19 variants in profile 1. Interestingly, previous work on BRAF, EGFR, and HER2 also focused on the length of the β3/αC loop as a determinant of both kinase activation in cancer and inhibitor sensitivity^59,60^. Deletions of five amino acids from the β3/αC loop are most common in EGFR exon 19 variants in lung cancer. Foster et al.^59^ also showed that shorter deletions can activate EGFR if a proline is introduced—presumably by restricting the β3/αC loop conformation. This may explain why L747P and ΔL747-A750InsP are activated and ΔL747-E749 is not, and why proline insertions (or substitutions, such as A750P, T751P, and S752P) make up ~25% of the list in Supplementary Fig. 10. Another more complex variant (ΔL747-K754InsATSPE) was also reported to show profile 1-like resistance^61^, with its β3/αC loop shortened by three residues and an inserted proline.

In the absence of crystal structures, much of the available structural information about exon 19 variants has been inferred from molecular dynamics (MD) simulations^12,62,63^. Significant discrepancies exist between the results of the different studies, however. In one such study^63^, the ΔE746-A750 TKD was reported to occupy a predominantly ‘αC-in’ conformation and to bind ATP more tightly than wild type EGFR—contrasting with our experimental findings. In another study, the ΔE746-A750 TKD was reported instead to have an ‘αC-out’ conformation in the deepest energy minimum^62^. Our own initial computational studies also suggested impaired erlotinib binding^12^, again not supported by the experimental data presented here. MD simulation results rely heavily on accurate initial protein structures, and these previous analyses will not have taken into account the different degrees of structural disorder that the individual variants show in their native states. Thus, our HDX-MS results provide an important dynamic perspective for considering ATP and TKI binding.

From a clinical perspective, another key observation from our studies is that profile 1 and profile 2 exon variants differ in their sensitivity to erlotinib and osimertinib, but not to afatinib (Figs. 1, 2a). This suggests that tumors driven by ΔL747-A750InsP, L747P or possibly other profile 1 exon 19 mutations, should respond better to afatinib than to first or third generation EGFR TKIs. Indeed, our previous cellular data suggest this for ΔL747-A750InsP^12^, and published in vitro and clinical data support this finding for L747P^27,64,65^. Why might profile 1 exon 19 variants show diminished inhibition by erlotinib and osimertinib but not by afatinib? For erlotinib, the increased ATP-binding affinity of profile 1 variants simply demands that more inhibitor (~10-fold more) is required to achieve the same level of inhibition seen with a profile 2 variant like ΔE746-A750 that binds ATP more weakly. For osimertinib, a covalent inhibitor, profile 1 mutations might weaken initial (reversible) binding either directly and/or through increased ATP competition. This would reduce the lifetime of the initial encounter complex and thus reduce the efficiency with which osimertinib reacts with C797 in the active site^66^. Alternatively (or in addition), the profile 1 mutations could alter the pose of osimertinib in the TKD’s ATP-binding site—as suggested for other EGFR mutations^67^—so that the reaction between the drug and C797 is impaired. These are the same mechanisms proposed to allow osimertinib to spare wild type EGFR in favor of the mutated receptor^31,67^. Reversible binding of afatinib to EGFR is substantially stronger than seen with osimertinib^31,66^, which should allow it to react more efficiently with profile 1 variants—and thus retain more complete inhibition—regardless of the specific exon 19 mutation. Although further detailed mechanistic study would be necessary to ascribe the retained afatinib sensitivity of profile 1 variants to enhanced affinity or reactivity, our data argue strongly that afatinib may be a more effective option for tumors with this class of uncommon exon 19 mutations.

In summary, our biochemical and structural analyses suggest that a simple classification of exon 19 variants based on β3/αC deletion length (and proline introduction) can provide a much-needed rationale to inform clinical decision-making for patients with tumors driven by uncommon exon 19 deletions, which is supported by analysis of patient outcome data.

## Methods

### Plasmid construction

For intact EGFR expression in CHO cells, cDNA for full-length EGFR (residues 1–1210 in precursor protein numbering—including the 24-residue signal sequence) in pcDNA3.1(−) was used (NCBI NP_005219.2, UniProt ID: P00533). EGFR mutations (L858R, T790M/L858R, ΔE746-A750, ΔL747-P753InsS, ΔL747-A750InsP, ΔE746-S752InsV, ΔL747-S752, ΔE746-T751InsA, ΔL747-T751InsP, ΔE746-T751, ΔL747-P753, ΔL747-E749, ΔS752-I759, L747P, ΔL747-T751InsS, ΔL747-T751InsQ, or ΔE746-S752InsI) were introduced into the wild type vector using QuikChange® site-directed mutagenesis (Agilent Technologies). For expressing the corresponding kinase domains, TKD sequences (spanning resides 696–1022 in precursor protein numbering or 672–998 in mature protein numbering) were amplified from the relevant EGFR cDNA in pcDNA3.1(−) using primers that introduced an N-terminal hexa-histidine (6x His) tag and Spe I/Xho I restriction sites for subcloning into pFastBac1. Recombinant baculoviruses were then generated using the Bac-to-Bac system (Invitrogen) for protein expression in *Spodoptera frugiperda* Sf9 cells (Expression Systems).

### Protein expression and purification

Sf9 cells at 1.6 to 1.8 × 10^6^ cells/ml were infected with recombinant baculovirus and cultured for 60–65 h at 27 °C. Cells were harvested by centrifugation at 2,200 × *g*, and cell pellets resuspended in cold (4 °C) lysis buffer (20 mM Tris/HCl, pH 8.0, containing 500 mM NaCl, 5 mM 2-mercaptoethanol, and 10% w/v glycerol—supplemented with Roche protease inhibitor cocktail). Cells were then lysed using a microfluidizer (Microfluidics M-110P). The cell lysate was centrifuged at 75,000 × *g* for 1 h to remove cell debris and insoluble aggregates, and the resulting supernatant was filtered through a 0.45 μm filter before loading onto a 5 ml HiTrap Chelating Ni^2+^ affinity column (Cytiva). The column was equilibrated in buffer A (20 mM Tris/HCl, pH 7.8, containing 500 mM NaCl), and EGFR TKD was eluted with buffer B (buffer A supplemented with 200 mM imidazole) using a step gradient over 20 column volumes. Pooled fractions were passed through a 0.22 μm syringe filter and further purified at 4 °C by gel filtration, using a HiLoad 16/60 Superdex 200 column (Cytiva) equilibrated in buffer C (20 mM Tris/HCl, pH 7.5, containing 250 mM NaCl, and 250 mM KCl). The purity and molecular weights were assessed by SDS-PAGE (Supplementary Fig. 1) imaged using a Bio-Rad GelDoc-EZ imager, running Image Lab Version 5.2.1, and intact mass spectrometry—with typical yields of purified EGFR TKDs ranging from 1.5 to 3 mg per liter of Sf9 culture.

### Steady-state kinase assays

All steady-state parameters for kinase activity and inhibition were determined using a quantitative fluorometric peptide assay that employed AssayQuant PhosphoSens® peptide substrate #0001 (AQpeptide), containing a sulfonamido-oxine (Sox) fluorophore that shows chelation-enhanced fluorescence upon peptide phosphorylation^28,29^. The assay reaction (20 μl) contained 50 mM HEPES (pH 7.5), 0.01% Brij-35, 10 mM MgCl_2_, 0.5% w/v glycerol, 0.1 mg/ml BSA, 1 mM DTT, and varying concentrations of AQpeptide—plus the noted concentration of purified recombinant EGFR TKD. Reactions were carried out in a 384-well assay plate format at 30 °C and initiated with the addition of AQpeptide after 5 min incubation of EGFR with ATP. Progress curves of phosphorylated peptide accumulation were monitored using a BioTek Synergy microplate reader with a fluorescence intensity module excitation and emission wavelength at 360 nm and 480 nm, respectively.

To convert relative fluorescence intensity readings to molar concentrations of phosphorylated AQpeptide, we fully phosphorylated AQpeptide at a range of different concentrations up to 200 μM by incubating for 1 h with 5 nM purified T790M/L858R variant (plus 1 mM ATP and 10 mM MgCl_2_) and plotted the fluorescence intensity counts of fully phosphorylated AQpeptide plotted against the known peptide concentration.

Michaelis-Menten constants (*K*_M_) of each EGFR variant for ATP (*K*_M, ATP_) were determined using 20 μM AQpeptide and a range of different ATP concentrations (0.98 μM–3 mM), with purified EGFR TKDs at the following concentrations: 5 nM (L858R/T790M), 10 nM (ΔL747-A750InsP), 50 nM (L858R), 100 nM (L747P, ΔL747-P753InsS, ΔS752-I759, ΔL747-T751InsP), 200 nM (ΔE746-T751InsA), 500 nM (ΔE746-A750), 1 μM (WT, ΔL747-E749), or blank (no EGFR). TKD concentrations were chosen to maintain steady-state kinetics for the first ~10 min of the reaction (See Supplementary Fig. 11). Initial velocities (*ν*_o_) at different ATP concentrations were determined by inspecting progress curves during the steady state phase. The *ν*_o_ values were plotted against ATP concentration, and the curves fit to the Michaelis-Menten equation to estimate *K*_M, ATP_ as well as *V*_max_ using GraphPad Prism. Similarly, *K*_M_ values of the EGFR TKD variants for AQPeptide (*K*_M, Peptide_) were estimated from studies at fixed ATP concentration (1 mM) by varying the concentration of AQpeptide substrate from 98 nM to 200 μM. Estimates for *k*_cat_ were also obtained from these experiments, using 1 mM ATP and saturating peptide. [ATP] in these experiments is more than 10 times *K*_M, ATP_ (i.e. saturating) for all EGFR variants except ΔE746-A750, ΔE746-T751InsA, and ΔL747-T751InsP, for which *k*_cat_ values may therefore be underestimates (noting that these variants may also not be saturated with peptide under these conditions).

### Determination of IC_50_ and inhibition constant (*K*_I_) for erlotinib

The half maximal inhibitory concentrations (IC_50_) of erlotinib, afatinib, and osimertinib were determined for each EGFR TKD variant by monitoring reaction progress curves at different concentrations of inhibitor in the presence of 1 mM ATP. Purified TKD at an estimated final concentration of 50–100 nM (10 times greater for ΔL747-E749) was mixed with 0 to 40 µM inhibitor (erlotinib, afatinib, or osimertinib) at 1 mM ATP (and 10 mM MgCl_2_) for 30 min. Reactions were initiated by adding 10 µM AQPeptide, and fluorescence intensity counts corresponding to phosphorylated AQpeptide were monitored using the BioTek Synergy 2 microplate reader. Initial velocities were determined as described above. IC_50_ values for each inhibitor were then determined by fitting the measured velocity (normalized to 100% in the absence of inhibitor) to the simple equation: Rate = 100/(1+[TKI]/IC_50_) using GraphPad Prism.

Inhibition constants (*K*_I_) for EGFR TKD variants were measured only for the reversible inhibitor erlotinib, and were determined by monitoring progress curves at different inhibitor concentrations in the presence of ATP. In 384-well format, EGFR TKD was mixed with different concentrations of inhibitor from 0 to 10 μM, and for each [TKI], samples with a series of ATP concentrations from 0.98 μM to 2 mM were tested. Reactions were initiated by adding 10 μM AQPeptide (20 μM for ΔL747-P753InsS), and fluorescence intensity counts resulting from AQPeptide phosphorylation were monitored using the BioTek Synergy 2 microplate reader. Initial enzyme velocities were determined as described above. Erlotinib IC_50_ values were then determined for each [ATP], and *K*_I_ was estimated by fitting these IC_50_ values to the Cheng-Prusoff Equation^34^:

$${{{{{{\rm{IC}}}}}}}_{50}={K}_{{{{{{\rm{I}}}}}}}\;(1+[{{{{{\rm{ATP}}}}}}]/{K}_{{{{{{\rm{M}}}}}},\;{{{{{\rm{ATP}}}}}}})$$

EGFR TKD concentrations for the different variants were: 5 nM (L858R/T790M), 10 nM (ΔL747-A750>P), 50 nM (L858R), 100 nM (L747P, ΔL747-P753InsS, ΔL747-T751InsP, ΔS752-I759), 200 nM (ΔE746-T751InsA), 500 nM (ΔE746-A750), 1 µM (ΔL747-E749), or blank (no EGFR).

### Mammalian cell lines and culture conditions

For routine cell culture, CHO cells (ATCC #CCL-61) were cultured in 100 mm cell culture dishes (Corning Falcon #353003) at 37 °C, in a humidified incubator with 5% CO_2_ in F-12K medium (Kaighn’s modification of Ham’s F-12 medium, ATCC #30-2004) supplemented with 10% fetal bovine serum (Gibco #16140-071) and penicillin (100 U/ml) and streptomycin (100 U/ml) (Gibco #15140122). Cells were obtained from ATCC, and routinely tested for mycoplasma contamination (Lonza #LT-07-118).

### Transient transfection and inhibitor treatment of CHO cells

For transfection, CHO cells were plated at a density of 2.0 × 10^5^ in 6-well plates (Corning Falcon #353046) in F-12K medium supplemented with 3% FBS and no antibiotics. The following day, FuGENE™ HD transfection reagent (Promega #PRE2311) was used to transfect each well with 0.7 µg of pcDNA3.1(−) containing the relevant EGFR variant. After 48 h, cells were starved using F-12K medium with no supplementation and treated after 72 h with erlotinib (SelleckChem #S7786), afatinib (SelleckChem #S1011) or osimertinib (SelleckChem #S7297) for 1 h at the noted concentrations. Inhibitors were made as 100× stocks in DMSO, and diluted to the final concentrations in starvation F-12K medium. After treatment, cells were placed on ice, medium aspirated, and 150 μl of ice-cold lysis buffer added per well. Lysis buffer was made from 10× stock (CST #9803) and supplemented with protease and phosphatase inhibitors (ThermoFisher #78440). Using a cell scraper (ThermoFisher #179693), lysates were harvested, vortexed, and centrifuged for 10 min at 10,000 × *g* at 4 °C. Protein concentrations in the clarified cell lysates were quantitated using a detergent-compatible protein assay (BioRad #5000111). Clarified cell lysates were combined with 4× Laemmli sample buffer (BioRad #1610747) containing a final concentration of 50 mM DTT, and heated for 4 min at 95 °C.

### Quantitative immunoblotting

Samples corresponding to 20 μg of total protein in clarified lysates were subjected to SDS-PAGE using 4–20% polyacrylamide stain-free gels (BioRad #4568096). Proteins were transferred onto 0.2 µm nitrocellulose membranes (BioRad #1620112). Membranes were blocked for 1 h using Intercept® TBS blocking buffer (LICOR #972-60001), washed extensively with TBS-T (10 mM Tris-HCl pH8.0, 150 mM NaCl, 0.1% w/v Tween20), and incubated with primary antibodies diluted in Intercept® T20 antibody diluent (LICOR #972-65001) overnight at 4 °C on an orbital shaker. The following primary antibodies were used (at 1:2000 dilution): phospho-EGFR (pEGFR) pY1173 (Rabbit, CST #4407), total EGFR (Mouse, ThermoFisher #MS-665-P0), Grb2 (Rabbit, CST #3972). Note that the EGFR pY1173 antibody recognizes phosphorylated Y1197 in the P00533 UniProt sequence. After extensive washing with TBS-T, blots were incubated with goat anti-mouse IRDye® 680RD (LICOR #926-68070) and/or goat anti-rabbit IRDye® 800CW (LICOR #926-32211) secondary antibodies at 1:20,000 dilutions for 1 h at room temperature. After final extensive washes with TBS-T, the 680RD total EGFR and 800CW pEGFR bands were simultaneously detected using a LICOR Odyssey® DLx imaging system. As in previous work^68,69^, Grb2 was used as a loading control because it is less abundant than housekeeping proteins such as actin, yielding more accurate normalization. For quantitation and plotting of Western blot data, the signal for each band was quantitated using Image Studio Version 5.2.5 (LICOR). Both total EGFR and pY1173 EGFR signals were normalized with respect to the Grb2 loading control. The pEGFR/total EGFR ratio was then plotted as a percentage of maximum receptor phosphorylation, seen in the DMSO-treated sample. Uncropped gels and confirmation of linearity in our detection of band intensity on the LICOR are shown in the Source data.

### Hydrogen–deuterium exchange mass spectrometry (HDX-MS)

Freshly purified EGFR exon 19 variant TKDs at 7.8–15.6 μM in 20 mM HEPES (pH 7.4), 100 mM NaCl-H_2_O buffer (5 μl) were manually labeled by 20-fold dilution with D_2_O-buffer (95% v/v final D_2_O concentration) containing 20 mM HEPES (pD 7.4), 100 mM NaCl at 25 °C. For deuterium labeling in the presence of erlotinib, each protein (7.8 μM to 15.6 μM) was pre-incubated with a 1.4- to 2-fold molar excess of erlotinib (15.6 to 20 μM). With all reported erlotinib *K*_I_ values being substantially less than 40 nM (~5 nM for proteins used for HDX here), >90% of the protein will be occupied by erlotinib under these conditions following 20-fold dilution with deuterium-containing buffer in the HDX experiment. The labeled samples were quenched at different time points (10, 60, 180, and 600 s) by adding 100 μl of cold 200 mM glycine buffer (pH 2.3). Similarly, the fully-deuterated samples were prepared by labeling the sample for 1 min with 20 mM HEPES (pD 7.4), 100 mM NaCl, in the presence of 8 M urea-d_4_ (Cambridge Isotope Laboratories, Inc.). Quenched samples were flash-frozen in liquid N_2_ and stored at −80 °C until mass spectrometry analysis. Frozen samples were quickly thawed and manually injected onto an Enzymate BEH pepsin column (Waters) at 2 °C, and the deuterium-labeled sample was digested for 3 min (at a flow rate of 100 μl/min water/0.1% formic acid). Peptic peptides were trapped and separated using an Acquity UPLC BEH C18, 130 Å, pre-column (2.1 × 5 mm, 1.7 μm, Waters catalogue number 186003975) and Acquity UPLC BEH C18, 130 Å, column (1.0 × 100 mm, 1.7 μm, Waters catalogue number 186002346), respectively, using a linear gradient from 5 to 40% acetonitrile over 7 min, on an Acquity UPLC M-Class w/HDX-2 automation (Waters), running at 40 μl/min. UPLC solvents were water containing 0.1% (v/v) formic acid (A) and acetonitrile containing 0.1% (v/v) formic acid (B). MS^e^ data were acquired using a Synapt G2-Si mass spectrometer (Waters) using 0.4 s scan time in sensitivity TOF mode. A ramp collision energy of 5 to 10 V was used for low energy acquisition, and 15 to 40 V for high energy acquisition with continuous lock mass (Leu-Enk) for mass accuracy correction. Other instrument parameters were: capillary voltage, 3 kV; cone voltage, 10 V; source offset, 80 V; source temperature, 90 °C; desolvation temperature, 150 °C; cone gas flow, 0 l/h; desolvation gas flow, 600 l/h; nebulizer gas, 6 bar; MS scan range, 300–2000 m/z.

### HDX-MS data analysis

Peptides were sequenced using the ProteinLynx Global Server 3.03 (PLGS, Waters), and the deuterium uptake of each peptic peptide was determined using DynamX 3.0 (Waters), with minimum intensity set to 5000, minimum product per amino acid to 0.3, and maximum MH^+^ error set to 10 ppm. The deuterium uptake of all analyzed peptides presented in this study is the average uptake of three biological samples (two for ΔL747-E749 and ΔL747-A750InsP) with each biological repeat performed in technical triplicate (each a separate labeling reaction). The percent exchange of each peptic peptide (%D) was calculated by the following equation:

$$\%{{{{{{\rm{Ex}}}}}}}=({m}_{t}-{m}_{0})/({m}_{f}-{m}_{0})\cdot 100$$

where *m*_*t*_ is the centroid mass of a peptic peptide at time, *t*; *m*_0_ is the centroid mass of a peptic peptide without deuterium labeling; and *m*_*f*_ is the centroid mass of a peptic peptide for the fully-deuterated standard sample. For plotting exchange data against residue number in Fig. 4a, the percent exchange for each peptide was plotted against the median residue number of the peptide. Mass spectra of peptides with EX1 exchange kinetics were analyzed and fit to a Gaussian distribution using Origin (OriginLab). All data were collected and analyzed according to consensus HDX-MS guidelines^70^, and are detailed the Source data file and Supplementary Table 1.

The DynamX software package (Waters) was used to map percent exchange data onto the structure of the EGFR TKD in Fig. 4b and Supplementary Fig. 7. In brief, for a residue within a given region, the percent exchange determined for the shortest peptide that includes it is assigned. For residues found in multiple overlapping peptides, the percent exchange value for a peptide that retains the overlapping region at its C-terminus is used.

### Determination of ΔL747-E749 EGFR TKD crystal structure

Purified EGFR TKD (∆L747-E749) was subjected to an additional anion exchange chromatography step^71^, concentrated to ~6 mg/ml in 20 mM Tris pH 8.0, 150 mM NaCl and 2% glycerol. Crystals were obtained using the hanging-drop vapor diffusion method with a reservoir solution of 28% PEG 400, 0.1 M HEPES pH 7.5, 0.2 M CaCl_2_ at 16 °C. Crystals appeared after two weeks and were cryo-protected in reservoir solution supplemented with 20% (w/v) glycerol and 5% (w/v) ethylene glycol prior to flash freezing in liquid nitrogen. X-ray diffraction data were collected on the synchrotron beamline 24-ID-C of NE-CAT at the Advanced Photon Source. Data were processed using XDS^72^ Version 20200417, and scaled using SCALA (Version 3.3.22) in the CCP4 program suite (Version 7.1)^73^. The structure was solved by molecular replacement using Phaser^74^. The active state EGFR TKD structure (PDB code 1M17^40^) was used as the search model. Repeated cycles of manual building/rebuilding were performed using Coot^75^ and were alternated with refinements using Phenix (Version 1.18.2_3874)^76^ and the PDB-REDO web server^77^ (https://pdb-redo.eu). The final structure was validated with the MolProbity (version 4.02b-467) and wwPDB (version 2.26) servers. Structural figures were generated using PyMOL (http://www.pymol.org).

### Clinical data analysis

De-identified patient data were obtained from the GENIE BPC non-small cell lung cancer v2.1 consortium database^54^, for patients with a diagnosis of NSCLC whose tumors harbored an EGFR exon 19 deletion. Only patients who received erlotinib and had outcome data for progression free survival (PFS) on erlotinib were included. Data were also obtained retrospectively from a cohort of patients with metastatic NSCLC harboring an EGFR exon 19 deletion who were treated with erlotinib at the Yale Cancer Center. All patients were enrolled in Institutional Review Board-approved protocols at Yale University School of Medicine. Ethics oversight was provided by the Yale University Institutional Review Board, and consent was obtained for use of patient clinical data.

For these analyses, PFS was defined as the time from treatment initiation to time of clinically significant growth of existing lesions or new lesions on imaging, or death. Overall survival (OS) was defined as time from treatment initiation to death. Profile 1 variants were defined as having deletions from the β3/αC loop of three or fewer residues, whereas profile 2 variants were defined as having β3/αC loop deletions of four or more residues. Profile 1 and profile 2 variants were grouped and compared using Kaplan-Meier survival analyses, using the Mantel–Cox test. Censoring occurred at the time of last clinical follow-up (denoted by an upward tick on the Kaplan–Meier plot). The Cox proportional hazards model^78^ was used to generate adjusted hazard ratios with 95% confidence intervals for PFS and OS, adjusting for baseline covariates of age, sex, race, and smoking history.

### Reporting summary

Further information on research design is available in the Nature Research Reporting Summary linked to this article.

## Supplementary information

Supplementary Info File #1

Reporting Summary

## Data Availability

The refined coordinates for the ΔL747-E749 EGFR TKD crystal structure have been deposited into the Protein Data Bank, with accession code PDB 7TVD. PDB entries 1M17, 7KXZ, and 3VJO were also used in generation of structural figures. HDX-MS mass spectrometry data have been deposited to the ProteomeXchange Consortium via the PRIDE^79^ partner repository, with the dataset identifiers; PXD037374 (ΔL747-A750InsP and L747P EGFR TKDs+/− erlotinib), PXD037355 (ΔE746-A750, ΔL747-P753InsS, and ΔL747-E749 EGFR TKDs+/− erlotinib), and PXD037448 (wild type EGFR TKD+/− erlotinib). Source data are provided with this paper as Source Data file. Uncropped gels and HDX-MS data are contained within the Source data file. Materials and correspondence requests should be addressed to Mark A. Lemmon (mark.lemmon@yale.edu), Kumar Ashtekar (kumar.ashtekar@yale.edu), or Yuko Tsutsui (yuko.tsutsui@yale.edu). Source data are provided with this paper.

## Source data

Source Data File
