# Supplementary material for: Biochemical and structural basis for differential inhibitor sensitivity of EGFR with distinct exon 19 mutations
Source: Nat Commun. 2022 Nov 10;13:6791. doi: 10.1038/s41467-022-34398-z (PMC9649653; doi:10.1038/s41467-022-34398-z)
Supplement: Supplementary file 1 — Supplementary Info File #1 [file 41467_2022_34398_MOESM1_ESM.pdf]

Supplementary Information

Biochemical and Structural Basis for Differential Inhibitor Sensitivity of EGFR with Distinct Exon 19 Mutations

Iris K. van Alderwerelt van Rosenburgh[1,2,3,§], David M. Lu[1,2,3,§], Michael J. Grant[3,4], Steven E. Stayrook[1,2,3], Manali Phadke[5], Zenta Walther[3,6], Sarah B. Goldberg[3,4], Katerina Politi[3,4,6], Mark A. Lemmon[1,2,3,#]*, Kumar D. Ashtekar[1,2,3]*, and Yuko Tsutsui[1,2,3]*

[1]Department of Pharmacology, Yale University School of Medicine, New Haven, CT 06520, U.S.A.

[2]Yale Cancer Biology Institute, Yale University West Campus, West Haven, CT 06516, U.S.A.

[3]Yale Cancer Center, Yale University School of Medicine, New Haven, CT 06520, U.S.A.

[4]Department of Medicine (Medical Oncology), Yale School of Medicine, New Haven, CT 06520, U.S.A.

[5]Yale Center for Analytical Sciences, Yale School of Public Health, New Haven, CT 06520, U.S.A.

[6]Department of Pathology, Yale University School of Medicine, New Haven, CT 06520, U.S.A.

§These authors contributed equally

#Lead contact

*Correspondence:yuko.tsutsui@yale.edu (Y.T.), kumar.ashtekar@yale.edu (K.D.A.), mark.lemmon@yale.edu (M.A.L.)

Supplementary Figures 1-11 and Supplementary Table 1

a

ΔE746-A750	3859	YES
ΔL747-P753InsS	331	YES
ΔL747-T751*	267	NO#
ΔL747-A750InsP	195	YES
ΔE746-S752InsV	142	NO*
ΔL747-S752	105	NO*
ΔE746-T751InsA	71	YES
ΔL747-T751InsP	48	YES
ΔE746-T751	39	NO*
ΔL747-P753	34	NO*
ΔL747-E749	33	YES
ΔS752-I759	32	YES
ΔL747P	27	YES
ΔL747-T751InsS	13	NO*
ΔL747-T751InsQ	8	NO*
ΔE746-S752InsI	4	NO*

b

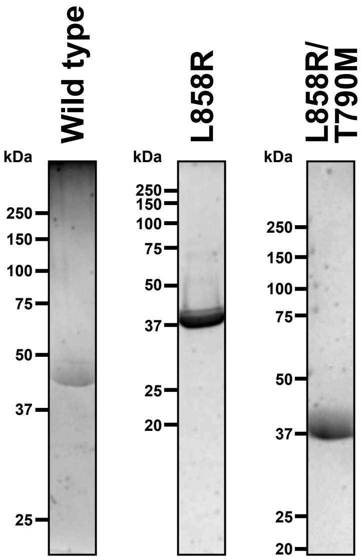

c

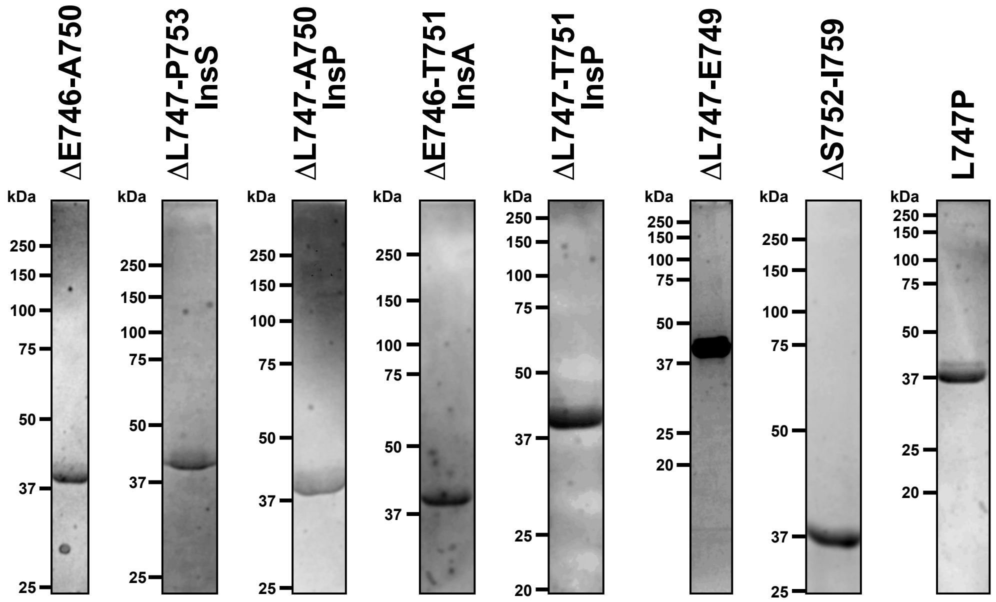

Supplementary Figure 1
Exon 19 mutants used for biochemical characterization.

a List of the fifteen most frequent rare exon 19 variants (plus ΔE746-A750), noting the number of times the variant is seen in the COSMIC database. We attempted to express and purify each of those listed as isolated TKDs. We failed to generate the expression construct for ΔL747-T751, and others designated 'NO' either did not express well in baculovirus-infected Sf9 cells or gave only aggregated protein. We successfully generated pure protein for eight of the variants, which are boxed in black and designated 'YES'. **b** We also generated the wild type EGFR TKD, and TKD variants harboring the L858R mutation or L858R/T790M double mutation. Coomassie stained SDS-PAGE gels of one representative preparation (of 5) of the corresponding purified proteins are shown. **c** Each accessible exon 19 variant was purified as isolated TKD and analyzed by Coomassie blue-stained SDS-PAGE (one representative preparation of 5 is shown). Experiments were not pursued if protein quality did not reach that of these preparations.

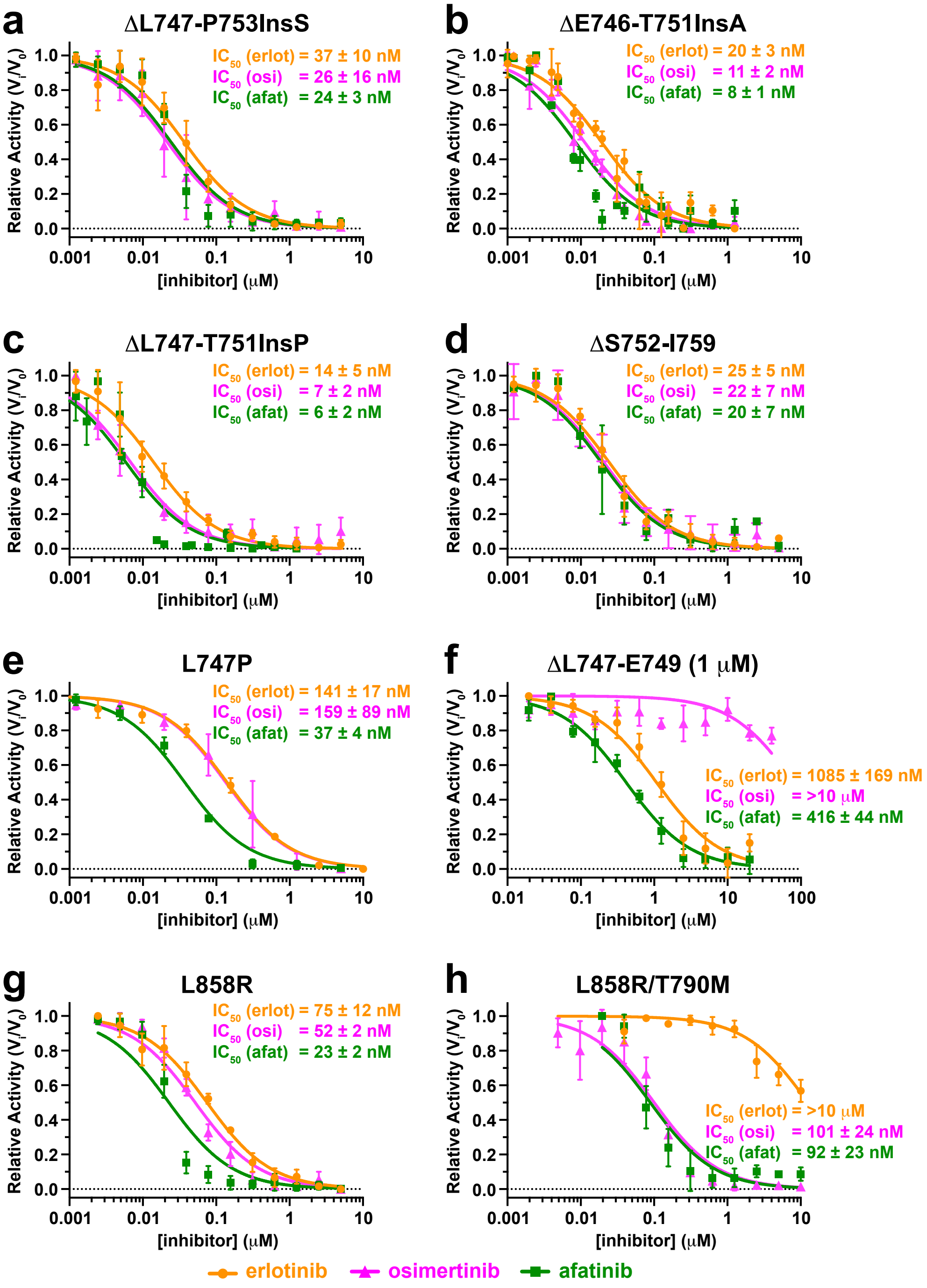

Supplementary Figure 2

Inhibition curves for EGFR TKDs harboring exon 19 and other mutations

a-h Concentration dependence of inhibition of the noted purified EGFR TKD variants by erlotinib (orange), afatinib (green) or osimertinib (magenta) in the presence of 1 mM ATP, 10 mM $MgCl_2$, and 10 μM peptide substrate at 30˚C (see Methods). Error bars on all points represent SD across replicates. IC_{50} values were obtained by fitting to the equation: Rate = $100/(1 + [TKI]/IC_{50})$, and mean IC_{50} values (± SD) are quoted for n = 3 separate protein preparations, with two independent experiments for each. TKD concentration was 100 nM for all but ΔL747-E749 (which has a low level of activity – see Table 1), instead analyzed at 1 μM.

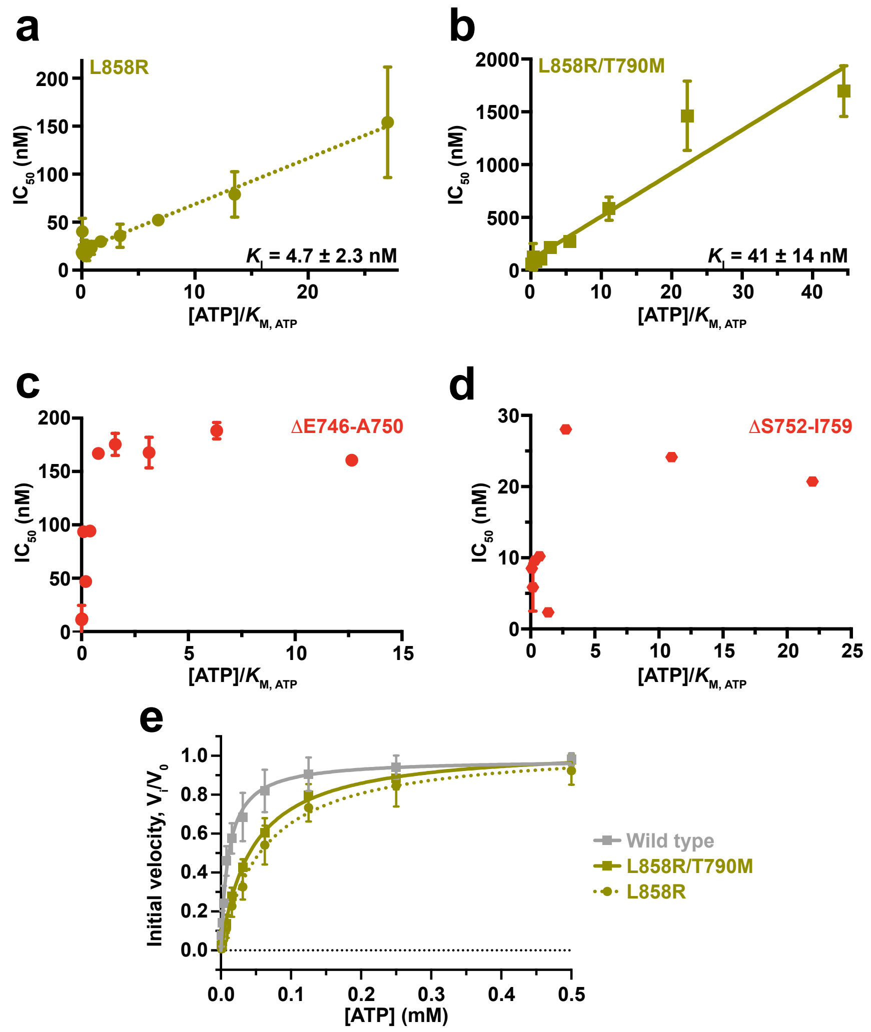

Supplementary Figure 3

Kinetic data for L858R variants, and Cheng-Prusoff plots for profile 2 exon 19 variants

a,b IC_{50} values for erlotinib were measured at a series of ATP concentrations for the L858R (**a**) and L858R/T790M (**b**) EGFR TKD, and plotted against the [ATP]/$K_{M, ATP}$ ratio for fitting to the Cheng-Prusoff equation to obtain K_I for erlotinib. Mean values (± SD) are plotted for n = 3 independent protein preparations. K_I is essentially the same for L858R as it is for ΔL747-A750InsP, L747P and ΔL747-P753InsS in Fig. 2c-e, with no significant difference ($P > 0.6$). By contrast L858R/T790M has a significantly higher K_I value than L858R ($P = 0.011$) and the exon 19 variants – suggesting a different means of promoting erlotinib resistance. P values are from unpaired two-sided Student's t-tests. **c, d** Equivalent experiments (plotting mean IC_{50} ± SD for three protein preparations) to determine the K_I for erlotinib with the ΔE746-A750 (**c**) and ΔS752-I759 (**d**) exon 19 variants, which did not yield reliable values. In these cases, IC_{50} appeared independent of [ATP]/$K_{M, ATP}$, suggesting some non-competitive inhibition and/or non specific binding that affects the erlotinib concentrations at which inhibition is achieved. **e** Michaelis-Menten plots for wild type EGFR TKD (black), L858R (olive circles) and L858R/T790M (olive squares), as in Fig. 2a. Mean initial velocity (± SD) is plotted against [ATP] for n = 3 separate protein preparations – with two independent experiments performed for each. $K_{M, ATP}$ values are given in Fig. 2b and Table 1.

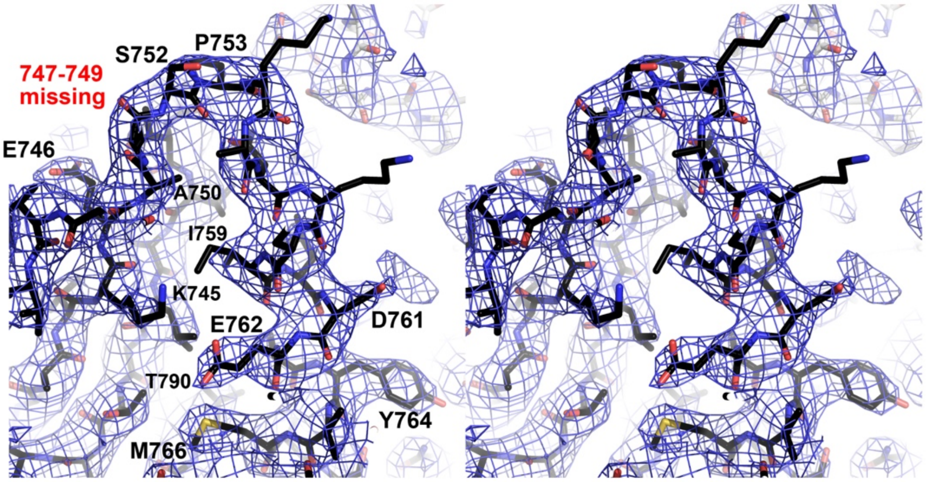

Supplementary Figure 4

Electron density in β3/αC region of ΔL747-E749 crystal structure

Stereo view of the 3 Å resolution ΔL747-E749 crystal structure reported here (PDB ID: 7TVD), showing the β3/αC region from which exon 19 deletion removes residues 747-749. E746 (labeled) is the last residue of strand β3, and αC begins at A755. Residues in the β3/αC loop and helix αC are marked, as are K745 in β3 and the αC glutamate E762. The density map, shown in blue mesh, is a 2Fo-Fc map contoured at 1.0 σ.

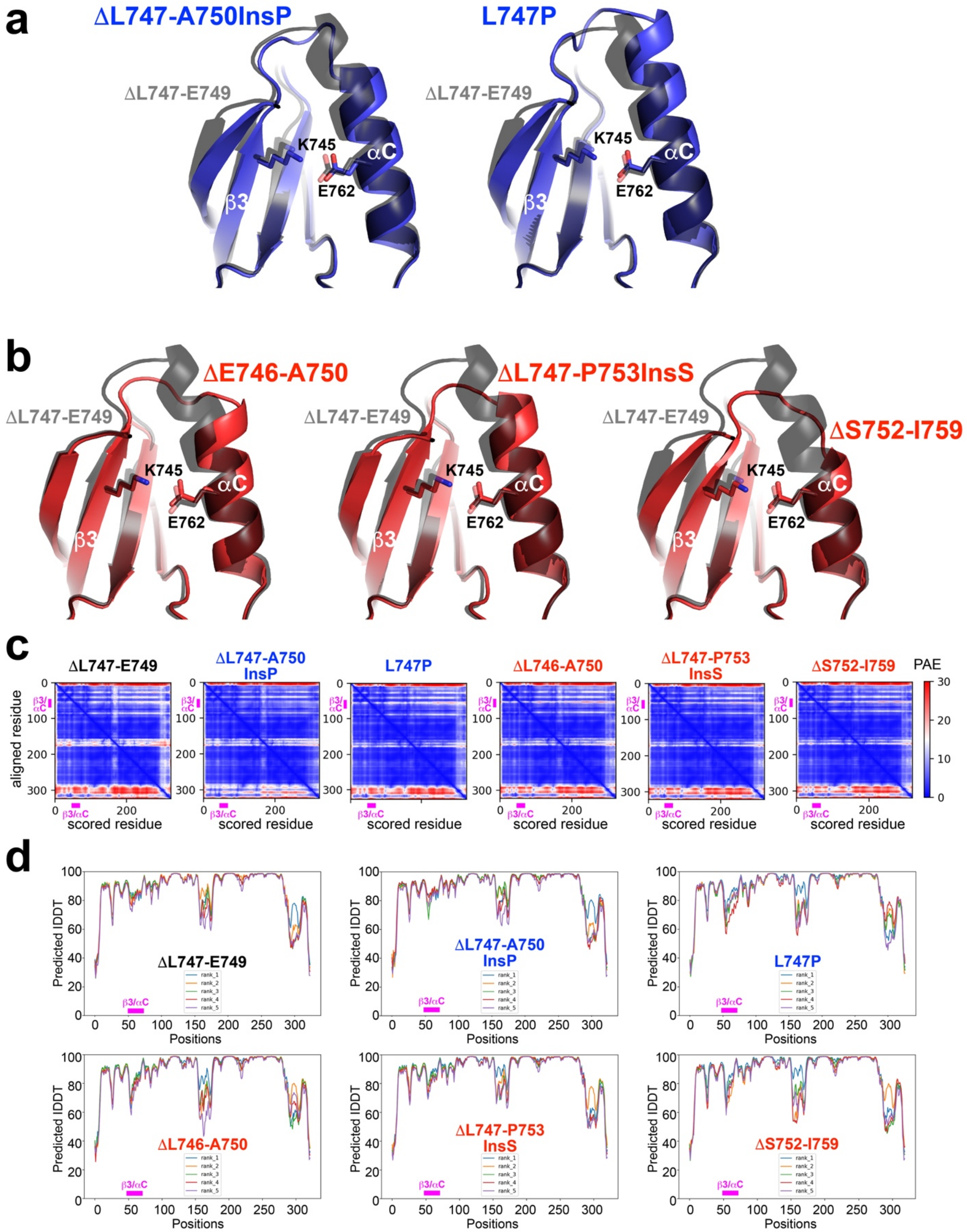

a
ΔL747-A750InsP
L747P
ΔL747-E749
K745
αC
E762
β3
b
ΔE746-A750
ΔL747-P753InsS
ΔS752-I759
c
ΔL747-E749
ΔL747-A750 InsP
L747P
ΔL746-A750
ΔL747-P753 InsS
ΔS752-I759
PAE
aligned residue
scored residue
β3/αC
d
Predicted lDDT
Positions
rank_1
rank_2
rank_3
rank_4
rank_5

Supplementary Figure 5

AlphaFold-based models for β3/αC region of EGFR TKD exon 19 variants

Structures of the noted exon 19 variants in profile 1 (**a**) and profile 2 (**b**) were modeled using ColabFold as described in the main text. In **a**, the profile 1 variant models are colored blue, and the AlphaFold-based model for ΔL747-E749 is shown in black with transparent cartoon. In **b**, the profile 2 variant models are colored red, with ΔL747-E749 black and transparent. **c** Plots of the 'predicted aligned error' or PAE for each model, showing AlphaFold's expected position error at residue x, when the predicted and true structures are aligned on residue y. The β3/αC loop position is marked in magenta. **d** Plots of predicted IDDT-C_α for each model, a per-residue measure of local confidence on a scale from 0 – 100. Residue 1 in the model corresponds to residue 696 in pro-EGFR.

Progressive deletion of residues from strand β3 and helix αC (marked) is accommodated in the models by shortening αC from its N-terminus, retaining the αC glutamate position (E762 in wild type) so that it can salt bridge with the β3 lysine (K745 in wild type) to stabilize ATP binding. The helix axis of truncated αC is also unchanged. We propose that the reduced αC length in profile 2 compared with profile 1 variants may explain the increased ATP-binding site flexibility/dynamics seen in our HDX-MS studies.

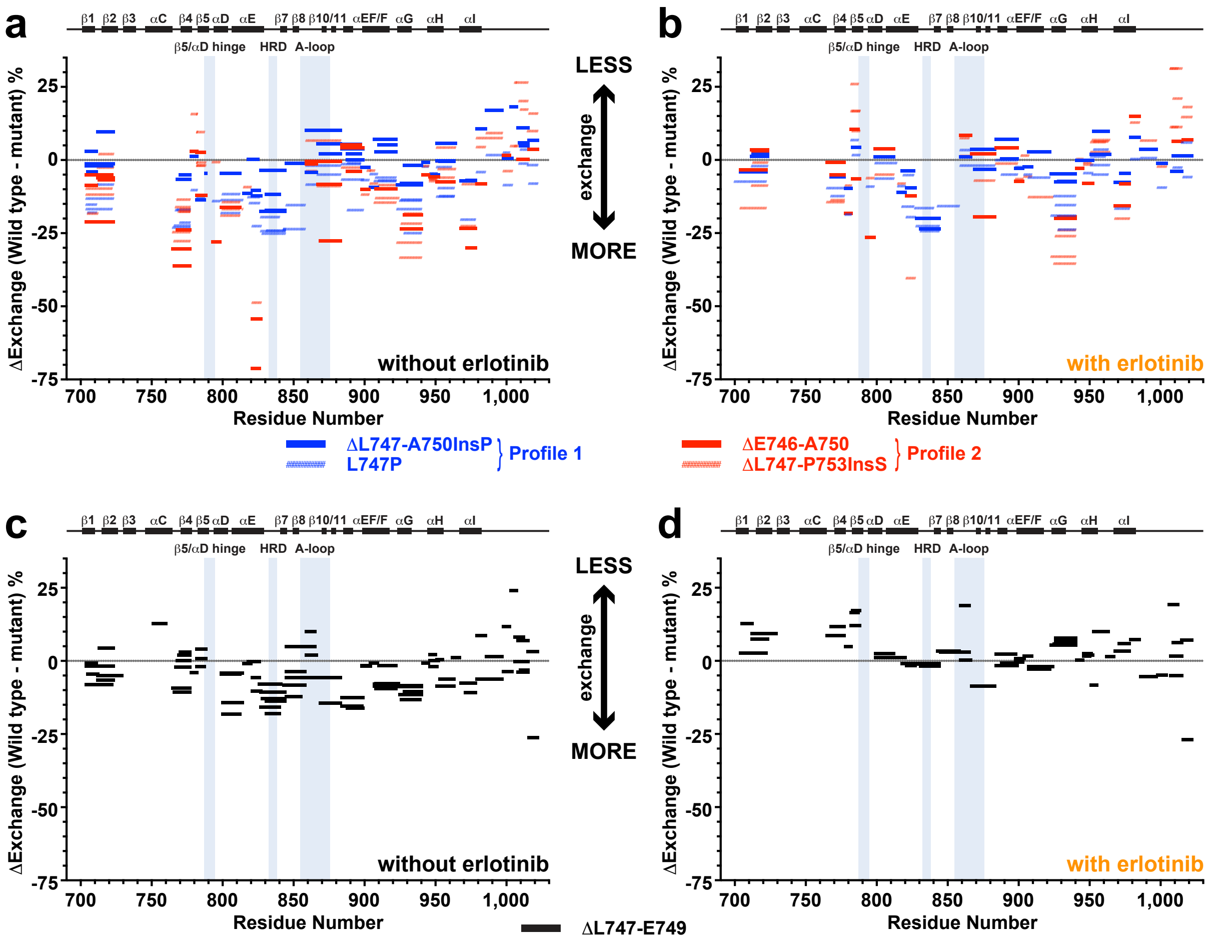

Supplementary Figure 6

HDX-MS percent exchange differences from wild type for exon 19 variants studied

Percent exchange difference (ΔExchange) for 1 min time point between wild type and each exon 19 variant shown for each peptide either without erlotinib (**a**,**c**) or when saturated >90% with erlotinib (**b**,**d**) as described in Methods. The black horizontal dotted line at ΔExchange = 0 represents wild type. In **a** and **b**, blue bars represent peptides from profile 1 exon 19 variants (ΔL747-A750InsP and L747P), and red bars represent those from profile 2 variants (ΔE746-A750 and ΔL747-P753InsS). In **c** and **d**, black bars represent peptides from ΔL747-E749. Positions of secondary structure elements are shown at the top of each panel. Peptides with positive ΔExchange values are in regions that show less exchange than wild type (i.e. become more stable than the corresponding regions in wild type). Peptides with negative ΔExchange values are in regions that show greater exchange than wild type (i.e. are structurally more flexible). Note that erlotinib addition (in **b** and **d**) generally reduces ΔExchange, indicating dampening of the overall dynamics of each exon 19 variant to become more similar to wild type. As shown in **c** and **d**, ΔL747-E749 is more generally similar to wild type than other exon 19 variants. Because mutations alter pepsin digestion patterns around mutated regions, the complement of peptides seen around β2, β3, and αC differs between wild type and mutants, so common peptides were not observed. ΔExchange values are also not shown for the binding pocket regions in ΔE746-A750 and ΔL747-P753InsS that showed EX1 exchange kinetics.

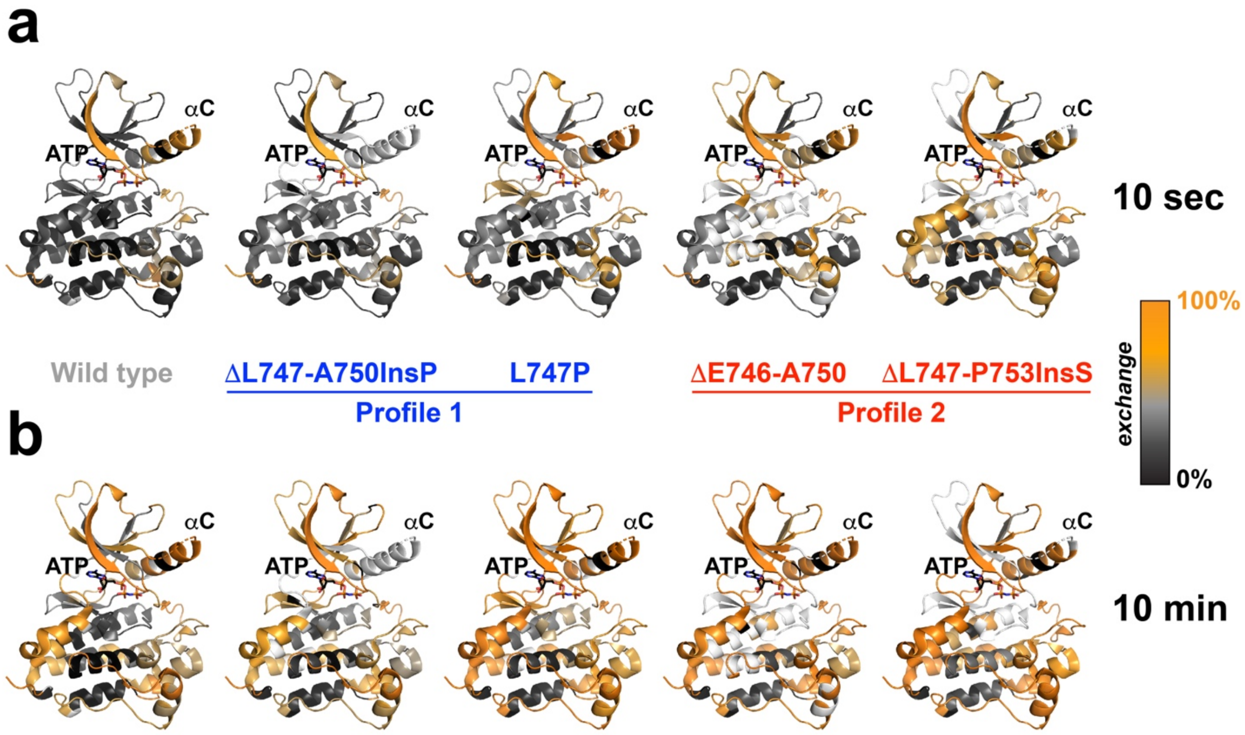

Supplementary Figure 7
Mapping of percent exchange onto EGFR-TKD structure for 10 s and 10 min HDX-MS data
Percent exchange values were assigned to each amino acid in the different EGFR-TKD values from HDX-MS data with labeling times of 10 s (**a**) and 10 min (**b**) using the DynamX software package (Waters) as described in *Methods* and Fig. 4b. These values were then mapped onto a crystal structure of the wild type EGFR TKD (PDB ID: 7KXZ) with ATP (sticks) placed using PDB ID 3VJO. The position of αC is indicated in each structure figure. Deuterium uptake values were corrected for back exchange and are expressed as percent exchange as described in *Methods*. As indicated by the scale at the right-hand site of the figure, orange-colored regions represent those with more exchange, suggesting more structural flexibility, whereas grey and black colored regions represent those with less exchange (and less flexibility). Regions that showed EX1 exchange kinetics (See main text, Fig. 6, and Supplementary Fig. 9) and regions without peptide coverage are colored white.

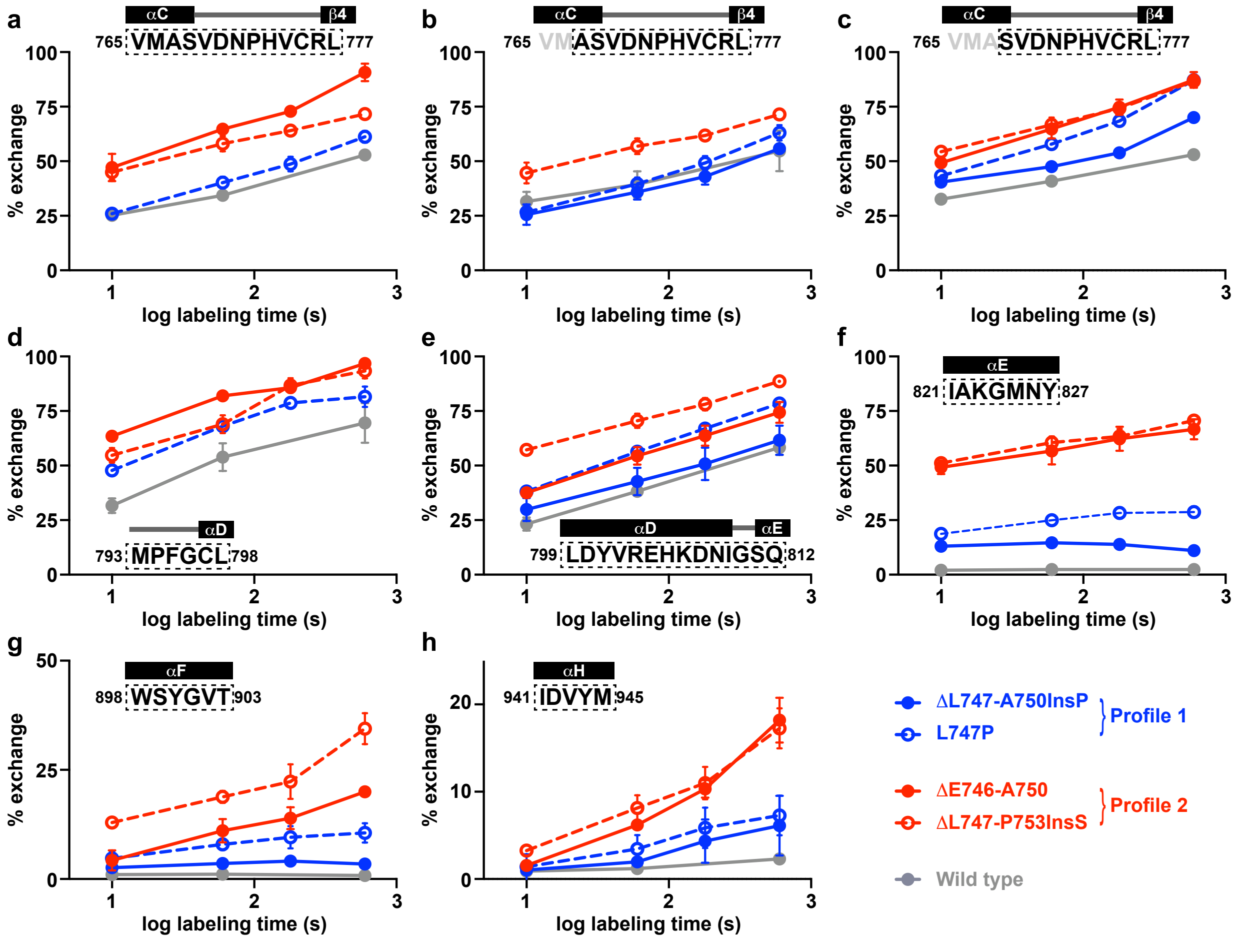

Supplementary Figure 8

Profile 2 variants show more backbone exchange than profile 1 in several areas

a-c Individual peptides around the αC/β4 loop of the TKD show increase exchange for the profile 2 ΔE746-A750 and ΔL747-P753InsS variants when detected than is seen for wild type (grey) or the ΔL747-A750InsP or L747P profile 1 variants. The same is true in the β5/αD hinge region (**d**). Finer differences are seen in αD itself (**e**), where ΔL747-P753InsS shows the greatest exchange, L747P (profile 1) and ΔE746-A750 appear equivalent to one another, and ΔL747-A750InsP resembles wild type. In helix αE, both profile 2 variants studied show substantially greater exchange than the profile 1 variants (**f**). This trend is also largely maintained in helix αF and αH (**g, h**). The amino acid sequence of the peptide is shown in each graph. Note that not all peptides were seen for all variants (and are not shown when this is that case). All points plotted represent the mean (± SD) from three independent experiments performed on each of two separate protein preparations.

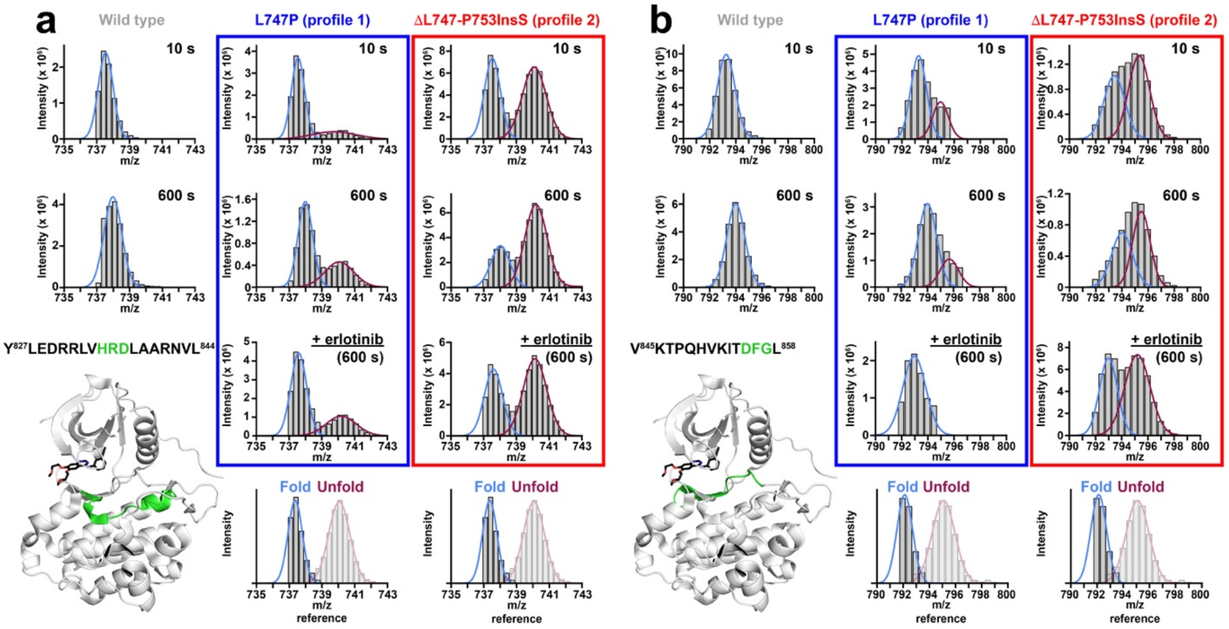

Supplementary Figure 9

The ATP-binding pocket of the profile 2 ΔL747-P753InsS variants is highly disordered

a Mass spectra of a triply charged peptide (MH^+ = 2209.2312 Da) containing the HRD motif in wild type (left), L747P (blue box), and ΔL747-P753InsS (red box) at the 10 s and 600 s labeling time points. The amino acid sequence of the peptide is shown above the crystal structure (bottom left) and colored green in the structure. The fully deuterated (unfolded) and no deuterium labeling (folded) reference mass spectra are shown below the erlotinib-bound mass spectra, as indicated. The areas below the light blue and red lines respectively in the bimodal mass envelopes correspond to the protein population with this peptide region (and environment) that is folded and unfolded. Note that erlotinib addition does not suppress the unfolded peak in ΔL747-P753Ins at the 600 s time point in the same way as seen in Fig. 6 for ΔE746-A750, whereas L747P closely resembles the (profile 1) ΔL747-A750InsP variant shown in Fig. 6. **b** Mass spectra of a doubly charged peptide (MH^+ = 1582.8952 Da) containing the DFG motif in wild type (left), L747P (blue box), and ΔL747-P753InsS (red box) at the 10 s and 600 s labeling time points. The peptide region is again colored green in the crystal structure at bottom left. Note that the bimodal distribution seen for ΔL747-P753InsS again does not 'collapse' into the folded population upon erlotinib addition as it did for ΔE746-A750 in Fig. 6b.

Variant	# in COSMIC
ΔE746	2
ΔL747	3
E746K/A/G/V/I/V	14
L747S/F/V	25
ΔE746-L747InsNY	1
ΔE746-R748	3
ΔE746-E749InsY	1
ΔE746-A750InsIP	9
ΔE746-A750InsRP	2
ΔE746-A750InsAP	3
ΔE746-A750InsVP	2
ΔE746-A750InsDP	1
ΔE746-A750InsQP	4
ΔE746-A750InsEP	2
ΔE746-A750InsGP	1
ΔE746-A750InsKP	1
ΔE746-T751InsAPS	1
ΔE746-T751InsFPS	1
ΔL747-R748	1
ΔL747-R748InsFP	1
ΔL747-E749InsC	1
ΔL747-E749InsP	1
ΔL747-A750InsS	1
ΔL747-A750InsC	1
ΔL747-A750InsE	1
R748K/I	7
E749G/V/K	4
A750P	12
A750/E/T/V	5
T751P	1
T751A/K/I/L	18
S752P	2
S752Y/F	9
P753S/L/F	28
K754E/A/R/I/Q	13
Total	182

Supplementary Figure 10
Unstudied profile 1 exon 19 variants

The COSMIC database contains an additional 57 exon 19 variants, listed here, that have not been studied biochemically or in patients, and would be predicted to be 'profile 1' variants (if activated) based on having 3 or fewer residues deleted from the $\beta 3/\alpha C$ loop. The variant is listed on the left, and the reported frequency of each variant in the COSMIC database on the right. These variants together account for ~3.5% of all exon 19 variants in COSMIC. Notably, 16 of these 57 short deletions and amino acid substitutions incorporate a non-native proline residue.

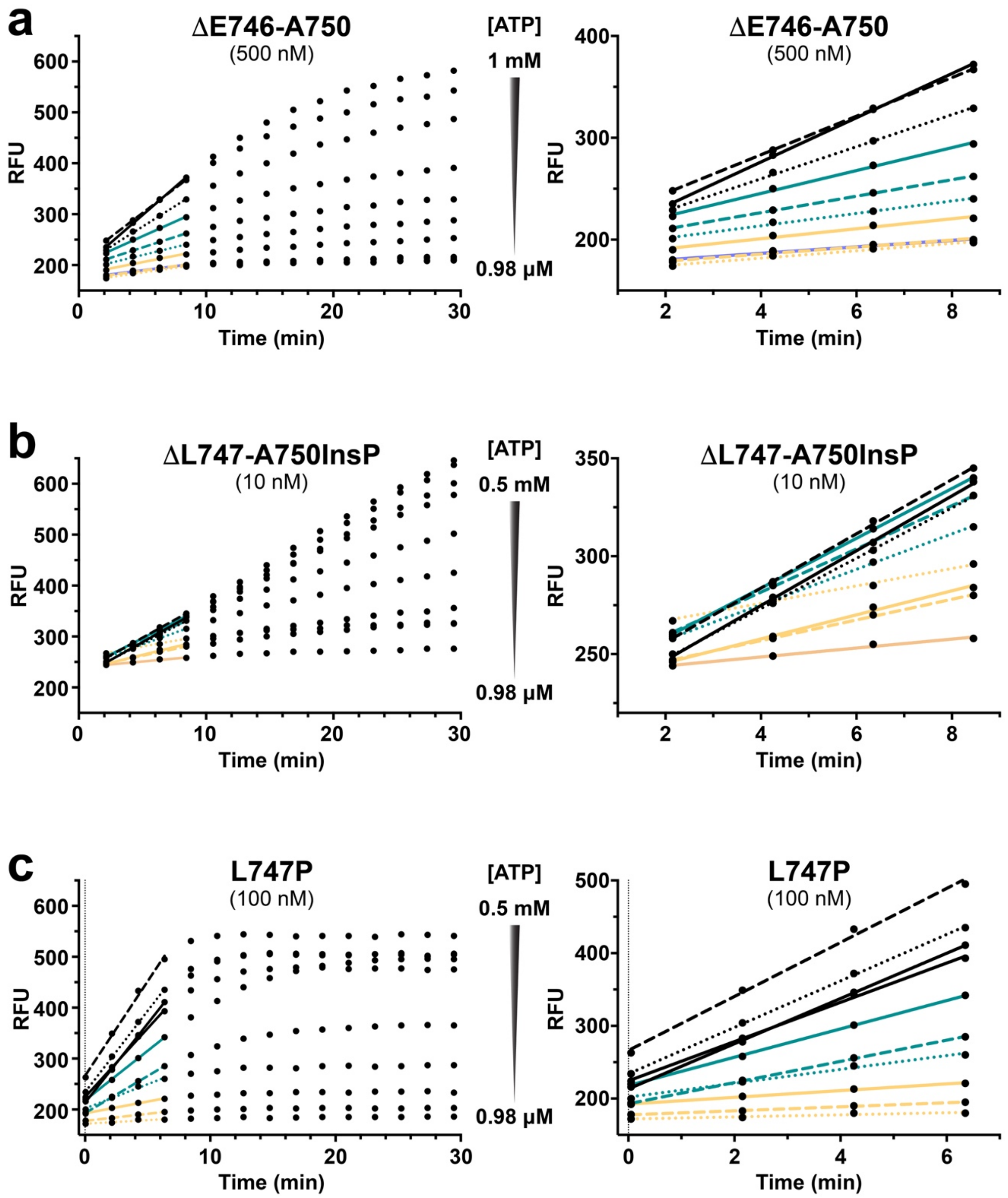

Supplementary Figure 11

Example progress curves for determining initial velocity (v_o)

The increase in the fluorescence emission at 485 nm (λ_{ex} = 360 nm) in the presence of the indicated exon 19 mutants was monitored at the noted enzyme concentration with varying [ATP] to measure the amount of the phosphorylated peptide product over time (see Methods). Conditions were optimized to ensure linearity, and example data are shown for key variants. Initial velocities were determined by taking the first derivative of the progress curves. The right-hand panels show expanded fitted linear regions used to determine initial velocities (see differently colored lines).

Supplementary Table 1
HDX-MS Summary Table

EGFR-TKD	ΔE746-A750	ΔL747-P753 InsS	ΔL747-A750 InsP	L747P	ΔL747-E749	wild-type
HDX reaction details	20 mM HEPES, 100 mM NaCl, pD 7.4, 25°C	20 mM HEPES, 100 mM NaCl, pD 7.4, 25°C	20 mM HEPES, 100 mM NaCl, pD 7.4, 25°C	20 mM HEPES, 100 mM NaCl, pD 7.4, 25°C	20 mM HEPES, 100 mM NaCl, pD 7.4, 25°C	20 mM HEPES, 100 mM NaCl, pD 7.4, 25°C
HDX time course (seconds)	0, 10, 60, 180, 600	0, 10, 60, 180, 600	0, 10, 60, 180, 600	0, 10, 60, 180, 600	0, 10, 60, 180, 600	0, 10, 60, 600, 3600, 7200
HDX control samples	Fully deuterated standard, labeled in 8 M urea-d_4, 20 mM HEPES, 100 mM NaCl, pD 7.4, 25°C	Fully deuterated standard, labeled in 8 M urea-d_4, 20 mM HEPES, 100 mM NaCl, pD 7.4, 25°C	Fully deuterated standard, labeled in 8 M urea-d_4, 20 mM HEPES, 100 mM NaCl, pD 7.4, 25°C	Fully deuterated standard, labeled in 8 M urea-d_4, 20 mM HEPES, 100 mM NaCl, pD 7.4, 25°C	Fully deuterated standard, labeled in 8 M urea-d_4, 20 mM HEPES, 100 mM NaCl, pD 7.4, 25°C	Fully deuterated standard, labeled in 8 M urea-d_4, 20 mM HEPES, 100 mM NaCl, pD 7.4, 25°C
Back-exchange (mean)	51.7%	47.7%	46.7%	49.6%	47.1%	47.4%
# of Peptides	41	86	62	73	115	138
Sequence coverage	85%	94%	83%	95%	96%	97%
Average peptide length/Redundancy	11.2/2.3	11.5/3.5	10.6/2.4	11.2/2.8	12.0/2.8	10.7/4.4
Replicates (biological, technical)	3 technical repeats on each of 3 separate protein preparations	3 technical repeats on each of 3 separate protein preparations	3 technical repeats on each of 2 separate protein preparations	3 technical repeats on each of 3 separate protein preparations	3 technical repeats on each of 2 separate protein preparations	3 technical repeats on each of 3 separate protein preparations
Repeatability (average standard deviation)	**No erlotinib:** 3.1% (0.15 Da) **+ erlotinib:** 2.7% (0.13 Da)	**No erlotinib:** 2.3% (0.12 Da) **+ erlotinib:** 2.9% (0.16 Da)	**No erlotinib:** 2.7% (0.15 Da) **+ erlotinib:** 2.1% (0.10 Da)	**No erlotinib:** 2.2% (0.11 Da) **+ erlotinib:** 2.6% (0.13 Da)	**No erlotinib:** 1.4% (0.08 Da) **+ erlotinib:** 0.9% (0.06 Da)	**No erlotinib:** 2.4% (0.12 Da) **+ erlotinib:** 1.8% (0.09 Da)
Significant differences in HDX	≥6.7% (0.31 Da) 98% CI	≥5.9% (0.32 Da) 98% CI	≥9.2% (0.5 Da) 92% CI	≥5.5% (0.28 Da) 98% CI	≥4.6% (0.27 Da) 92% CI	≥4.9% (0.24 Da) 98% CI
