# Supplementary material for: Biochemical and structural basis for differential inhibitor sensitivity of EGFR with distinct exon 19 mutations
Source: Nat Commun. 2022 Nov 10;13:6791. doi: 10.1038/s41467-022-34398-z (PMC9649653; doi:10.1038/s41467-022-34398-z)
Supplement: Supplementary file 2 — Reporting Summary [file 41467_2022_34398_MOESM2_ESM.pdf]

nature portfolio

Corresponding author(s): Mark A. Lemmon
Kumar D. Ashtekar
Yuko Tsutsui

Last updated by author(s): Oct 15, 2022

Reporting Summary

Nature Portfolio wishes to improve the reproducibility of the work that we publish. This form provides structure for consistency and transparency in reporting. For further information on Nature Portfolio policies, see our Editorial Policies and the Editorial Policy Checklist.

Statistics

For all statistical analyses, confirm that the following items are present in the figure legend, table legend, main text, or Methods section.

n/a	Confirmed	
☐	☒	The exact sample size (*n*) for each experimental group/condition, given as a discrete number and unit of measurement
☐	☒	A statement on whether measurements were taken from distinct samples or whether the same sample was measured repeatedly
☐	☒	The statistical test(s) used AND whether they are one- or two-sided<br>*Only common tests should be described solely by name; describe more complex techniques in the Methods section.*
☐	☒	A description of all covariates tested
☐	☒	A description of any assumptions or corrections, such as tests of normality and adjustment for multiple comparisons
☐	☒	A full description of the statistical parameters including central tendency (e.g. means) or other basic estimates (e.g. regression coefficient) AND variation (e.g. standard deviation) or associated estimates of uncertainty (e.g. confidence intervals)
☐	☒	For null hypothesis testing, the test statistic (e.g. *F*, *t*, *r*) with confidence intervals, effect sizes, degrees of freedom and *P* value noted<br>*Give P values as exact values whenever suitable.*
☒	☐	For Bayesian analysis, information on the choice of priors and Markov chain Monte Carlo settings
☒	☐	For hierarchical and complex designs, identification of the appropriate level for tests and full reporting of outcomes
☒	☐	Estimates of effect sizes (e.g. Cohen's *d*, Pearson's *r*), indicating how they were calculated

Our web collection on statistics for biologists contains articles on many of the points above.

Software and code

Policy information about availability of computer code

Data collection	Crystallographic data were collected at the Advanced Photon Source (APS) NE-CAT @ APS beamline, 24-ID-C. Mass spectrometry data were acquired using a Synapt G2-Si mass spectrometer (Waters). Western blot images were collected using near-IR fluorescence detection and a LICOR Odyssey DLx imaging system. Coomassie stained gels were imaged using a Bio-Rad GelDoc-EZ imager, running Image Lab Version 5.2.1. Kinase assays were performed by detecting fluorescence with a BioTek Synergy 2 plate reader in house.
Data analysis	Crystallographic datasets were integrated using XDS Version 20200417, and scaled using SCALA (Version 3.3.22) from the CCP4 program suite (Version 7.1). Structures were solved by molecular replacement using Phaser (Version 2.8.3), and model building performed using Coot (in CCP4 7.1), with repeated cycles of manual building/rebuilding performed using Coot, alternated with refinements using Phenix (Version 1.18.2_3874) and the PDB-REDO web server (https://pdb-redo.eu). Final structures were validated with the MolProbity (version 4.02b-467) and wwPDB (version 2.26) servers. For HDX-MS, peptides were sequenced using the ProteinLynx Global Server 3.03 (PLGS, Waters), and the deuterium uptake of each peptic peptide was determined using DynamX 3.0 (Waters). Structural figures were generated using PyMol Version 2.0.7. Enzyme kinetic data were analyzed, and data graphs drawn using GraphPad Prism 9 for macOS (Version 9.2.0). Western blot images were quantitated using Image Studio (LICOR), Version 5.2.5.

For manuscripts utilizing custom algorithms or software that are central to the research but not yet described in published literature, software must be made available to editors and reviewers. We strongly encourage code deposition in a community repository (e.g. GitHub). See the Nature Portfolio guidelines for submitting code & software for further information.

Data

Policy information about availability of data

All manuscripts must include a data availability statement. This statement should provide the following information, where applicable:

- Accession codes, unique identifiers, or web links for publicly available datasets
- A description of any restrictions on data availability
- For clinical datasets or third party data, please ensure that the statement adheres to our policy

The refined coordinates for theL747-E749 EGFR TKD crystal structure have been deposited into the Protein Data Bank, with accession code PDB 7TVD [http://doi.org/10.2210/pdb7TVD/pdb]. PDB entries 1M17, 7KXZ, and 3VJO were also used in generation of structural figures. Source Data for gels and HDX-MS data are provided with this paper.
HDX-MS mass spectrometry data have been deposited to the ProteomeXchange Consortium via the PRIDE79 partner repository, with the dataset identifiers:
PXD037374 (ΔL747-A750InsP and L747P EGFR TKDs +/- erlotinib); [http://proteomecentral.proteomexchange.org/cgi/GetDataset?ID=PXD037374]
PXD037355 (ΔE746-A750, ΔL747-P753InsS, ΔL747-E749 EGFR TKDs +/- erlotinib); [http://proteomecentral.proteomexchange.org/cgi/GetDataset?ID=PXD037355]
PXD037448 (wild type EGFR TKD +/- erlotinib); [http://proteomecentral.proteomexchange.org/cgi/GetDataset?ID=PXD037448]

Human research participants

Policy information about studies involving human research participants and Sex and Gender in Research.

Reporting on sex and gender	Clinical data are from retrospective analysis of de-identified patient data.
Population characteristics	De-identified patients with a diagnosis of NSCLC whose tumors harbored an EGFR exon 19 deletion.
Recruitment	N/A - this was not a clinical trial
Ethics oversight	Yale University Institutional Review Board

Note that full information on the approval of the study protocol must also be provided in the manuscript.

Field-specific reporting

Please select the one below that is the best fit for your research. If you are not sure, read the appropriate sections before making your selection.

☒ Life sciences ☐ Behavioural & social sciences ☐ Ecological, evolutionary & environmental sciences

For a reference copy of the document with all sections, see nature.com/documents/nr-reporting-summary-flat.pdf

Life sciences study design

All studies must disclose on these points even when the disclosure is negative.

Sample size	Sample sizes were not predetermined based on statistical methods, but were chosen according to the standards of the field (at least three independent experiments, with independent protein preparations) and precedent of ours and others previous studies (e.g. PMC7543951, PMC4269829, and PMC3319004).
Data exclusions	No data were excluded.
Replication	At least three independent experiments were performed for each study, as detailed in the legends, using 2-3 separate protein preparations. All attempts are replication were successful with the exception of cases where no signals were detected because of technical problems.
Randomization	Randomization is generally not used for biochemical and biophysical studies of this sort, and it is not clear how it would be implemented for these in vitro studies in which characteristics of different variants are being measured with quantitative methods.
Blinding	Blinding is not applicable for this type of study, in which quantitative methods are used to measure characteristics of different variants. There was also no expected result for any of the variants going into the study.

Reporting for specific materials, systems and methods

We require information from authors about some types of materials, experimental systems and methods used in many studies. Here, indicate whether each material, system or method listed is relevant to your study. If you are not sure if a list item applies to your research, read the appropriate section before selecting a response.

Materials & experimental systems

n/a	Involved in the study
☐	☒ Antibodies
☐	☒ Eukaryotic cell lines
☒	☐ Palaeontology and archaeology
☒	☐ Animals and other organisms
☒	☐ Clinical data
☒	☐ Dual use research of concern

Methods

n/a	Involved in the study
☒	☐ ChIP-seq
☒	☐ Flow cytometry
☒	☐ MRI-based neuroimaging

Antibodies

Antibodies used

CST #4407 - Rabbit monoclonal IgG against EGFR pY1173, clone 53A5 (used at 1:2,000 dilution for Western blotting)
MS-665-P0 - Mouse monoclonal IgG1 against total human EGFR, clone H9B4 from ThermoFisher (used at 1:2,000 dilution for Western blotting)
CST #3972 - Rabbit polyclonal against Grb2 (used at 1:2,000 dilution for Western blotting)
LICOR #926-68070 - goat anti-mouse IgG IRDye® 680RD (used at 1:20,000 dilution as secondary antibody for Western blotting)
LICOR #926-32211 - goat anti-rabbit IgG IRDye® 800CW (used at 1:20,000 dilution as secondary antibody for Western blotting)

Validation

All antibodies used are commercially purchased and have been validated by the vendors. Validation data are available from the respective vendor's respective websites. In addition, inclusion of well characterized positive and negative controls in our experiments (for EGFR activation) - in this and previously published studies - serves to validate each antibody for its use in monitoring these signaling events. Specifically, anti-EGFR was confirmed to give no signal in EGFR knock-out cells, and antibodies to pEGFR were shown to give signal only when well known activators of EGFR were added (cell starvation minimizing signal). We have previously used the primary antibodies extensively in a recent publication (PMC7521189).

Specific validation information:

1. EGFR pY1173, clone 53A5 (https://www.cellsignal.com/products/primary-antibodies/phospho-egf-receptor-tyr1173-53a5-rabbit-mab/4407) - validated by manufacturer for: Species;human and Application; Western blotting.

2. EGFR (total), clone H9B4 (https://www.fishersci.com/shop/products/epidermal-growth-factor-receptor-egfr-ab-15-mouse-monoclonal-antibody-epredia/p-4541942) - validated by manufacturer for: Species;human and Application; Western blotting.

3. Grb2 - polyclonal antibody - (https://www.cellsignal.com/products/primary-antibodies/grb2-antibody/3972) - validated by manufacturer for: Species;hamster and Application; Western blotting.

4. IgG IRDye® 680RD secondary antibody - (https://www.licor.com/bio/reagents/irdye-680rd-goat-anti-mouse-igg-secondary-antibody) - validated by manufacturer for: Species;mouse IgG and Application; Western blotting.

5. IgG IRDye® 800CW secondary antibody (https://www.licor.com/bio/reagents/irdye-800cw-goat-anti-rabbit-igg-secondary-antibody) - validated by manufacturer for: Species;rabbit IgG and Application; Western blotting.

Eukaryotic cell lines

Policy information about cell lines and Sex and Gender in Research

Cell line source(s)	CHO cells (#CCL-61) and Sf9 (CRL-1711) cells were purchased from ATCC .
Authentication	Cells were not re-authenticated after receipt from (trusted) source.
Mycoplasma contamination	Confirmed negative with routine testing (Lonza #LT-07-118).
Commonly misidentified lines (See ICLAC register)	None
